# Causality of circulating vitamins on infectious diseases: integrating Mendelian randomization and *in vivo* evidence

**DOI:** 10.3389/fimmu.2025.1674678

**Published:** 2025-12-01

**Authors:** Aling Tang, Zhimin Gong, Yi Shi, Weizhen Zhang, Mingming Jin, Lichao Sun, Wei Chen

**Affiliations:** 1 Longhua Hospital Affiliated to Shanghai University of Traditional Chinese Medicine, Shanghai, China; 2 Shanghai University of Medicine & Health Sciences, Shanghai, China; 3 Department of Emergency, China-Japan Friendship Hospital, Beijing, China

**Keywords:** infection, vitamin, bacteria, virus, sepsis

## Abstract

**Background:**

Clinical studies have established an association between infections and circulating vitamin levels. However, the relationship stratified by specific pathogen types remains underexplored. More importantly, the causal direction of this association is still unclear.

**Methods:**

We utilized summary-level data from genome-wide association studies (GWAS) of European ancestry, sourced from the UK Biobank (UKB) and FinnGen consortium. A two-sample, bidirectional Mendelian randomization (MR) analysis was employed to investigate the genetic causal relationships between infectious diseases (categorized as bacterial or viral) and circulating levels of vitamins A, B6, B12, C, D, 25-hydroxyvitamin D (25(OH)D), and E. The inverse variance weighted (IVW) method served as the primary analytical approach. Sensitivity analyses were conducted to validate the robustness of the findings. Furthermore, we employed the cecal ligation and puncture (CLP) model in mice to assess the impact of sepsis on serum 25(OH)D levels.

**Results:**

Genetically predicted higher circulating vitamin E levels were associated with an increased risk of viral infection (OR = 1.45, 95% CI: 1.10–1.88). Conversely, genetic predisposition to bacterial infection was associated with lower circulating 25(OH)D levels (OR = 0.96, 95% CI: 0.93–0.99). *In vivo* experiments confirmed a significant decrease in serum 25(OH)D levels in CLP group mice compared to the Sham group (Sham: 90.7 ± 1.7 vs. CLP: 47.9 ± 5.6). No causal relationships were identified between infections and other vitamins.

**Conclusion:**

This study provides evidence for a potential causal link between elevated vitamin E levels and increased susceptibility to viral infection, as well as between bacterial infection and reduced 25(OH)D levels. Furthermore, our *in vivo* data demonstrated that sepsis (induced by intraperitoneal bacterial infection) led to a significant decrease in serum 25(OH)D levels.

## 1 Introduction

Infectious diseases, caused by pathogenic microorganisms such as bacteria, viruses, and fungi, pose a significant global health challenge. This challenge is exacerbated by the increasing rates of antimicrobial resistance, which threaten the efficacy of existing treatments (1, 2). Vitamins, as essential micronutrients, have garnered widespread attention for their potential roles in modulating infectious diseases (3). Their physiological functions are complex and may involve immunoregulation, anti-inflammatory effects, and antioxidant activities (4). Consequently, vitamin supplementation has been explored as a potential adjuvant therapy for infections (5, 6). However, evidence regarding the efficacy of vitamins remains inconsistent. Some studies indicate that supplementation with specific vitamins is not associated with a reduced risk of infections and may even correlate with increased mortality in certain infectious contexts (7, 8). Mendelian randomization is an analytical method that uses genetic variants strongly associated with a phenotype—typically single nucleotide polymorphisms (SNPs)—as instrumental variables to infer causal relationships between the phenotype and disease risk. By integrating summary-level data from large-scale genome-wide association studies, effect estimates are obtained for the associations between these genetic instruments and the exposure (e.g., vitamin levels), as well as for their associations with the outcome (e.g., infectious disease risk). Subsequently, MR analysis methods such as the inverse-variance weighted approach are applied to estimate the causal effect of a per-unit change in genetically predicted serum vitamin levels on the outcome risk. Under the satisfaction of key instrumental variable assumptions, this method effectively mitigates reverse causality and confounding biases commonly encountered in observational studies (9). Leveraging this approach, our study employed a two-sample, bidirectional MR analysis to investigate the causal links between circulating levels of multiple vitamins and infectious diseases stratified by pathogen type.

## 2 Methods

### 2.1 Study design

We performed a two-sample bidirectional MR analysis to assess the causal relationships between circulating vitamin levels and infectious diseases. The analysis included vitamins A, B6, B12, C, D, 25-hydroxyvitamin D (25(OH)D), and E. Infectious diseases were categorized into bacterial and viral infections for investigation. Three assumptions must be met in the MR Analysis: Specifically, the relevance assumption (genetic instruments are strongly associated with the exposure) was ensured by selecting SNPs that reached genome-wide significance. The independence assumption (instruments are independent of confounders) was addressed by using genetic variants from large, population-based GWAS of European ancestry. The exclusion restriction assumption (instruments affect the outcome only through the exposure) was evaluated through multiple sensitivity analyses to detect and correct for horizontal pleiotropy (9).

### 2.2 Data sources

The summary-level data for circulating vitamin B6, B12, C, D, and E levels were derived from a genome-wide association study (GWAS) conducted within the UK Biobank (UKB), comprising 64,979 individuals of European ancestry. The GWAS summary statistics for 25-hydroxyvitamin D (25(OH)D) were obtained from a larger UKB cohort of 417,580 European participants (10), while those for vitamin A were sourced from an independent GWAS of 8,247 Europeans (11). Data for infectious diseases were acquired from the FinnGen consortium. Bacterial and viral infection cases were defined based on international classification codes (ICD-8, ICD-9, ICD-10: A20-A49, B25-B34), encompassing 44,934 cases and 332,343 controls for bacterial infections, and 9,805 cases and 367,472 controls for viral infections. To minimize population stratification bias, all datasets were restricted to individuals of European ancestry. As this study utilized exclusively published and publicly available summary statistics, no additional ethical approval was required.

### 2.3 Selection of instrumental variables

Genetic variants associated with the exposures at genome-wide significance (p < 5 × 10^-6^) were selected as candidate instrumental variables (IVs). To ensure independence among the IVs, we clumped these single-nucleotide polymorphisms (SNPs) using a linkage disequilibrium (LD) threshold of r² < 0.001 within a 10,000 kb window, based on the 1000 Genomes European reference panel. The strength of each instrument was quantified using the F-statistic, calculated as F = β²/SE². All retained SNPs exhibited F-statistics substantially greater than 10, thereby effectively mitigating potential bias from weak instruments (12). These rigorously screened SNPs were subsequently employed as the final IVs in the Mendelian randomization analyses.

### 2.4 Cecal ligation and puncture model

Male C57BL/6J mice (6–8 weeks old) were obtained from Shanghai Slaughter Laboratory Animal Co., Ltd. A total of 12 mice were used in this study, randomly assigned to either the CLP group (n=6) or the sham-operated control group (n=6). All animals were maintained in a specific pathogen-free (SPF) facility under controlled conditions (temperature: 22 ± 2°C; 12 h light/dark cycle) with ad libitum access to food and water (1 week).

All animal handling procedures were conducted strictly in accordance with the regulations of the P.R. China on the use and care of laboratory animals and were approved by the Animal Ethics Committee of Shanghai University of Medicine & Health Sciences (Approval No: 2015-16-340406198707142817).

Sepsis was induced via cecal ligation and puncture as follows: Mice were anesthetized using inhaled isoflurane. The abdominal area was shaved and disinfected with alternating applications of iodine and 75% alcohol. A 1-1.5 cm midline incision was made to expose the cecum, which was gently exteriorized using a sterile cotton swab. The distal 50-75% of the cecum was ligated with 4–0 silk suture and punctured once through-and-through with a 21-gauge needle. A small amount of fecal material was extruded by gentle pressure before the cecum was returned to the abdominal cavity. The incision was closed in layers using 5–0 vicryl suture. Sham-operated mice underwent identical procedures except for cecal ligation and puncture.

Post-operative care included immediate fluid resuscitation (1 mL pre-warmed saline administered subcutaneously), sustained warmth provision, and analgesia (buprenorphine, 0.05 mg/kg). Animal welfare was monitored every 6 hours for the first 48 hours until the study endpoint. The course of sepsis in mice was detected from six aspects: appearance, consciousness level, activity, response to stimuli, eyes and respiratory quality. At 12 hours post-procedure, mice were euthanized via cervical dislocation under deep anesthesia (5% isoflurane) for sample collection.

### 2.5 25(OH)D

Blood was collected 12 hours after CLP, left at room temperature for 2 hours, and then centrifuged to obtain serum, which was stored at -80°C. The measurement of mouse serum 25(OH)D levels was performed according to the reagent manufacturer’s instructions (Shanghai Enzyme-linked Biotechnology, YJ3403B).

### 2.6 Statistical analysis

All statistical analyses were performed using R software (version 4.3.1). We applied the inverse variance weighted (IVW), MR-Egger, and weighted median methods to estimate the causal relationships in our two-sample MR framework. Among these, the IVW method was prioritized as the primary analytical approach due to its superior statistical power under valid instrumental variable assumptions (13). A statistically significant association derived from the IVW method was considered indicative of evidence for causality, provided it was not contradicted by the results of other MR methods.

To evaluate the robustness of the findings, we conducted comprehensive sensitivity analyses. Heterogeneity across genetic variants was assessed using Cochran’s Q statistic, and the random-effects model was applied irrespective of its presence (14). Horizontal pleiotropy was examined via the MR-Egger intercept test (15) and the MR-PRESSO global test (16). Outlier variants identified by MR-PRESSO and RadialMR (17) were removed, and the MR analysis was repeated to obtain corrected effect estimates. The MR-PRESSO distortion test was used to determine if the causal estimates differed significantly before and after outlier removal. The leave-one-out sensitivity test provides results before the exclusion of outliers. The results were visually inspected using funnel plots to assess symmetry and scatter plots to visualize the effect estimates. Forest plots, along with funnel and scatter plots based on the dataset after outlier exclusion, are provided in the **supplementary materials**.

Statistical analysis was performed using GraphPad Prism version 9.0. Data are presented as mean ± standard deviation. An independent-samples T-test was used to compare the two groups. Statistical significance was set at P < 0.05.

## 3 Results

### 3.1 Circulating vitamin levels and viral infectious diseases

**Figure 1:**
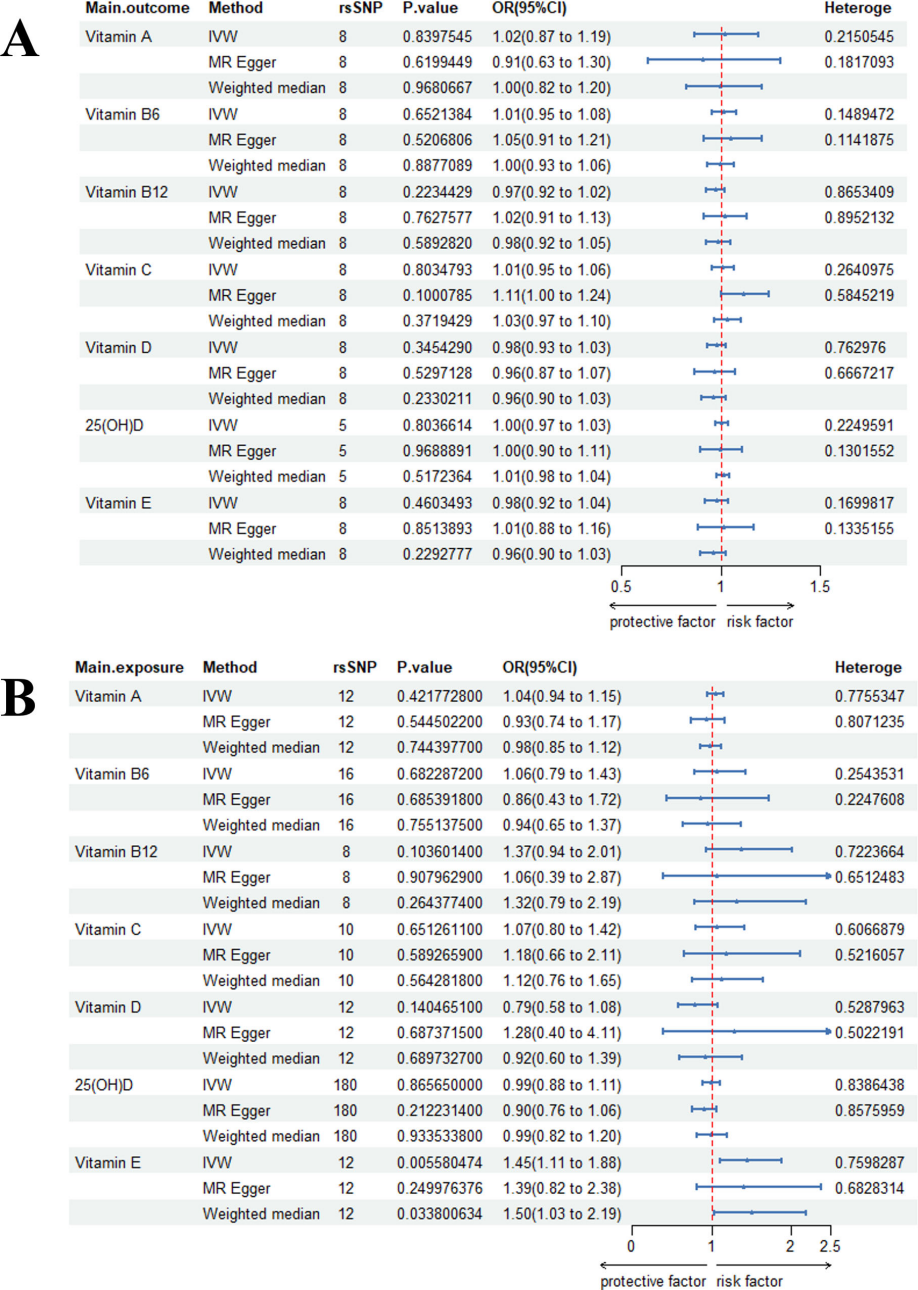
Bidirectional causal estimation of circulating vitamin levels and viral infection. **(A)** Viral infection is an exposure; **(B)** Viral infection is the outcome.

The strength of all instrumental variables was confirmed, with mean F-statistics exceeding the threshold of 10 (**Supplementary Material 1**). Detailed information on all IVs is provided in the same supplement.In the primary Mendelian randomization analysis, genetically predicted higher circulating vitamin E levels demonstrated a positive causal association with the risk of viral infectious diseases (OR = 1.45, 95% CI: 1.10–1.88, P = 0.0056; **Figure 1**). No significant causal relationships were observed for other vitamins in relation to viral infections. Reverse MR analyses, which treated viral infections as the exposure, also yielded non-significant results.Sensitivity analyses indicated potential horizontal pleiotropy for 25-hydroxyvitamin D (25(OH)D) via the MR-Egger intercept test (**Supplementary Material 2**). Subsequent outlier detection using MR-PRESSO and RadialMR identified several influential SNPs for 25(OH)D (**Supplementary Materials 3 & 4**). After removing these outliers, the repeated MR analysis produced consistent results, confirming the robustness of the initial findings. Leave-one-out sensitivity analysis further verified that no single SNP was driving the causal estimates (**Supplementary Material 5**). While some funnel plots suggested the presence of heterogeneity (**Supplementary Material 6**), the overall results remained stable. Visual representations of the MR associations are provided in scatter plots (**Supplementary Material 7**).

### 3.2 Circulating vitamin levels and bacterial infectious diseases

The mean F-statistic for all instrumental variables (IVs) exceeded 10 (**Supplementary Material 1**), indicating that the genetic instruments were robust against weak instrument bias. Detailed information on all IVs is provided in **Supplementary Material 1**.

**Figure 2:**
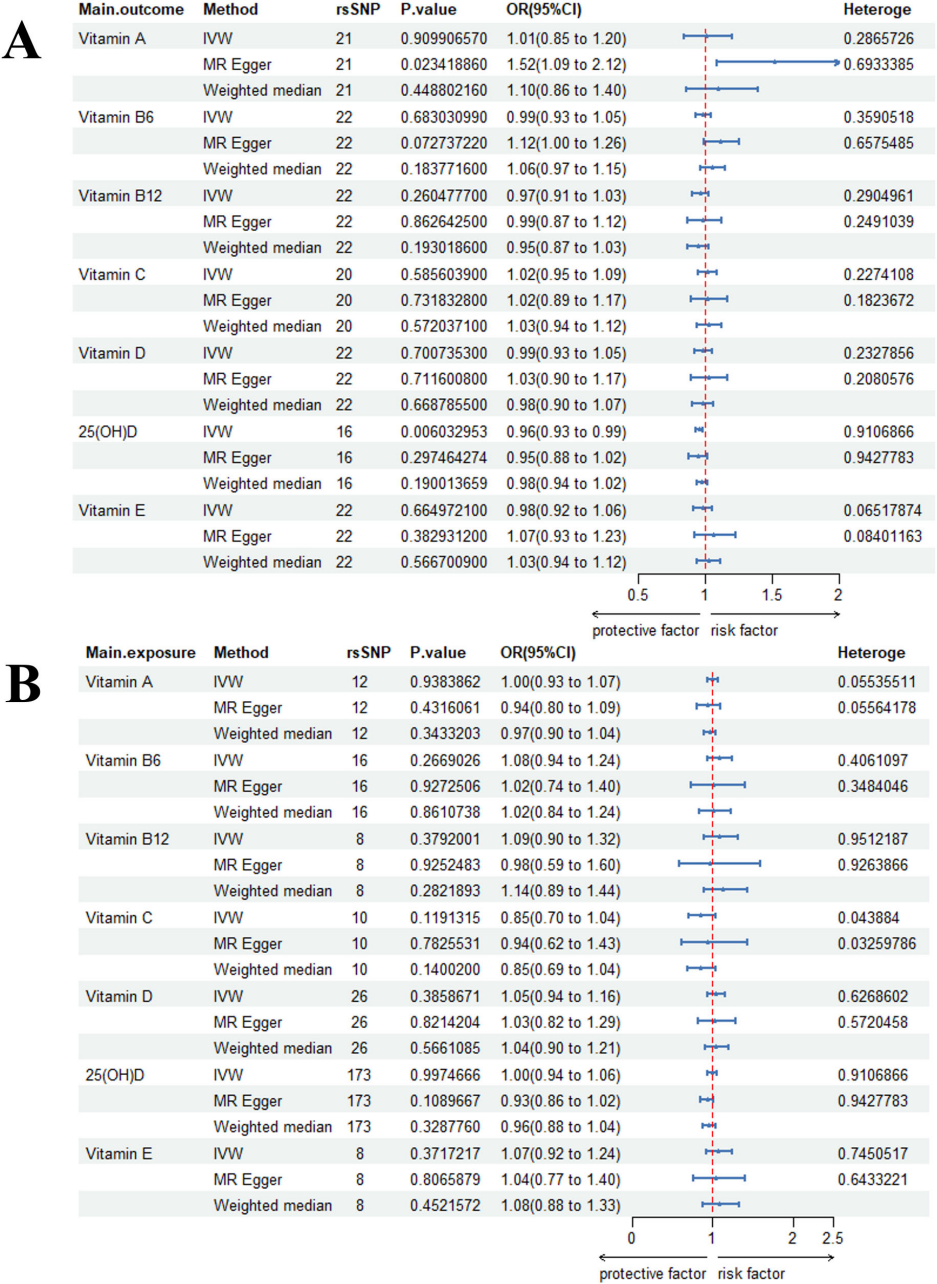
Bidirectional causal estimation of circulating vitamin levels and Bacterial infection. **(A)** Bacterial infection is an exposure; **(B)** Bacterial infection is the outcome.

In the primary MR analysis conducted prior to outlier removal, no significant causal associations were detected. However, after excluding outlier SNPs, a significant causal effect of genetic predisposition to bacterial infection on lower circulating 25(OH)D levels was identified (OR = 0.96, 95% CI: 0.93–0.99, P = 0.006; **Figure 2**).

Evidence of horizontal pleiotropy was observed for vitamin A and vitamin B6 when bacterial infection was set as the exposure, as indicated by the MR-Egger intercept test (**Supplementary Material 2**). Outlier SNPs were detected using MR-PRESSO and RadialMR methods (**Supplementary Material 3**), with specific outliers identified for 25(OH)D and vitamin E (when bacterial infection was the outcome), as well as for 25(OH)D and vitamin C (when bacterial infection was the exposure) (**Supplementary Material 4**). Notably, the leave-one-out sensitivity analysis performed prior to outlier removal suggested that the initial causal estimate for the effect of 25(OH)D on bacterial infection was driven by a single SNP (rs429358) (**Supplementary Material 5**). This influential SNP was subsequently identified and removed as an outlier by the MR-PRESSO procedure. Some funnel plots indicated the presence of heterogeneity (**Supplementary Material 6**). Scatter plots visually summarizing the MR associations are provided in **Supplementary Material 7**.

### 3.3 *In vivo* validation

**Figure 3:**
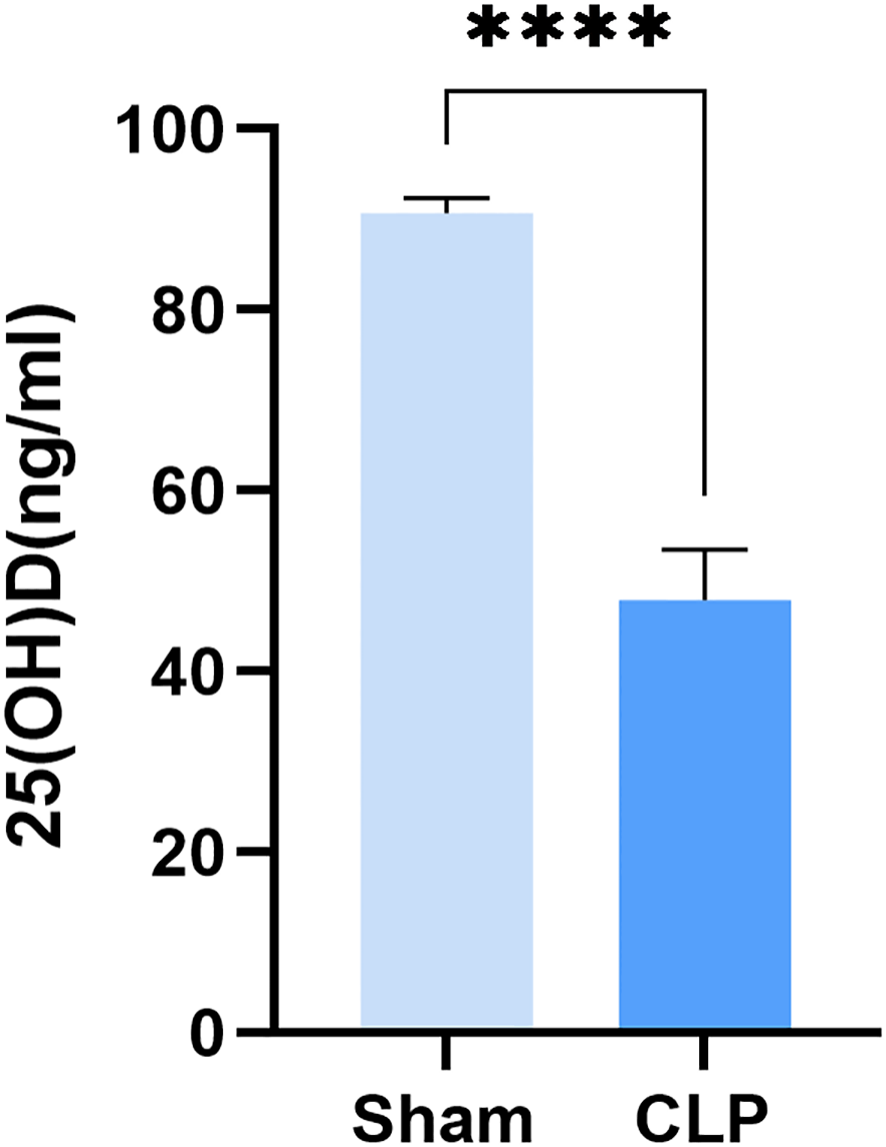
The effect of CLP on serum 25(OH)D levels in mice. **** means P < 0.0001.

The CLP model, a classic method for establishing sepsis via intra-abdominal bacterial infection, was utilized. Serum levels of 25(OH)D were measured 12 hours post-surgery, which revealed a significant decrease in the CLP group compared to Sham group (**Figure 3**).

## 4 Discussion

In this bidirectional two-sample Mendelian randomization study, we investigated the causal relationships between seven circulating vitamins and infections caused by bacteria or viruses. Our analysis demonstrated a positive causal effect of genetically predicted circulating vitamin E levels on the risk of viral infectious diseases. Conversely, we found that genetic predisposition to bacterial infection exerted a negative causal effect on circulating 25(OH)D levels. No robust causal associations were observed for the other vitamins examined.

Vitamins, as essential trace elements, have garnered extensive attention for their roles in modulating infections. Previous studies have reported that vitamin A deficiency is associated with an increased prevalence of respiratory infections among children and adolescents (18). While vitamin A supplementation has been shown to enhance immune function in deficient individuals (19) and reduce the incidence of respiratory infections in this population (20), some evidence suggests that high-dose supplementation may increase the risk of acute respiratory infections in normal-weight children (21). This discrepancy may be attributed to variations in infection types and host immune responses (22). Similarly, preclinical studies indicate that vitamin B6 alleviates sepsis-induced oxidative stress in the lungs and liver (23). Notably, our Mendelian randomization analysis did not support a causal relationship between vitamin A or B6 levels and bacterial or viral infections. For both vitamins in the context of bacterial infection, we detected evidence of horizontal pleiotropy, although no outlier SNPs were identified. Consequently, the null findings in these specific analyses should be interpreted with caution, as the underlying genetic variants may influence the outcomes through pathways independent of the vitamin exposures.

Vitamin B12, a member of the B vitamin complex, is recognized for its role in immune regulation and antiviral activity (24). However, evidence regarding its clinical impact remains inconsistent. One study reported that supplementation with vitamin B12 and folic acid did not reduce the incidence of diarrhea or lower respiratory tract infections and was associated with an increased risk of persistent diarrhea (25). Aligning with this, our MR analysis found no causal association between genetically predicted vitamin B12 levels and the risk of infectious diseases. The role of vitamin C in infection has been more widely studied than that of other vitamins. As infections often induce oxidative stress, vitamin C is frequently investigated as an antioxidant therapeutic agent. Interestingly, its effects appear highly heterogeneous and may be beneficial only under specific conditions or in particular patient subgroups (26). This heterogeneity is reflected in conflicting clinical findings. For example, one randomized controlled trial in 872 patients with sepsis found that intravenous vitamin C administration was associated with a higher risk of 28-day mortality and persistent organ dysfunction compared to placebo (27). In contrast, another study demonstrated significant reductions in in-hospital and 90-day mortality among sepsis patients who received intravenous vitamin C for ≥5 days (28), while other research observed no effect on key prognostic outcomes (29). Consistent with the ambiguous clinical evidence, our MR study did not support a causal relationship between vitamin C levels and susceptibility to bacterial or viral infections.

Vitamin D has also been extensively studied in the context of infection. It exerts immunomodulatory functions and contributes to host defense against bacterial and viral pathogens (30). While some studies suggest that vitamin D supplementation protects against acute respiratory infections (31, 32), and lower circulating 25(OH)D levels—the major form of vitamin D—have been associated with higher risk of such infections (33), other reports do not support a protective role of supplementation against SARS-CoV-2 infection, severe COVID-19, or other acute respiratory outcomes (34, 35). In line with the latter, our MR analysis did not support a causal effect of vitamin D on viral infection risk.A notable finding from our study is that genetic predisposition to bacterial infection was causally linked to lower circulating 25(OH)D levels. This observation provides mechanistic support for the clinical practice of vitamin D supplementation in patients with bacterial infections. However, reverse MR analyses did not indicate that vitamin D or 25(OH)D influences the risk of infectious diseases. Thus, our results do not support the use of vitamin D supplementation for preventing infectious diseases in the general population—a conclusion consistent with several earlier studies (34, 35). Sepsis is a life-threatening systemic inflammatory response triggered by various pathogens and remains a major clinical challenge. Among these, bacterial infections are a significant cause of sepsis (36). The CLP-induced model of sepsis (intra-abdominal bacterial infection) is a common method for studying this condition. In our CLP model, serum 25(OH)D levels were strikingly decreased, providing experimental evidence for the clinical correlation between bacterial infection and vitamin D status. The significant association between infection and 25(OH)D levels may be related to immune responses and liver-mediated vitamin D metabolism. During infection, immune cells increase their utilization of 25(OH)D to meet the demands of local immune defense, which may lead to an overall reduction in systemic circulating 25(OH)D levels (37). CYP2R1 is a key enzyme in the liver responsible for hydroxylating vitamin D into 25(OH)D (38). In cases of severe infection (such as sepsis), which can cause impaired liver function (39), the decline in 25(OH)D levels may result from the downregulation of CYP2R1 and CYP27A1 (another 25-hydroxylase), coupled with the upregulation of the catabolic enzyme CYP24A1 (40, 41).

A substantial body of literature indicates that under deficient or normal conditions, vitamin E acts as an effective antioxidant and enhances immune function (42). The effects of vitamin E may vary depending on individual characteristics and lifestyle factors. For instance, among individuals with the highest levels of smoking exposure and physical inactivity, vitamin E supplementation has been associated with an increased risk of pneumonia (43–45). Studies have also revealed sex-specific differences in the effects of vitamin E. Specifically, vitamin E intake was positively correlated with the incidence of upper respiratory tract infections in men. In studies related to HIV infection, higher pre-infection vitamin E levels were linked to increased mortality. Mendelian randomization analyses, which are less susceptible to confounding factors such as sex and lifestyle, suggest that elevated circulating vitamin E levels may contribute to a higher risk of viral infectious diseases. We propose that these highly heterogeneous outcomes may be attributed to the importance of antioxidant balance. An excessively strong antioxidant environment could inadvertently interfere with certain oxidative signaling pathways necessary for the immune system to clear viruses. For example, an effective T-cell response against viruses requires moderate levels of reactive oxygen species as signaling molecules (46). Supraphysiological antioxidant levels—such as extremely high vitamin E concentrations—may blunt these critical immune alarm and killing signals. This implies that the relationship between vitamin E and health may not be linear. Maintaining a balanced, rather than maximized, antioxidant state is likely most beneficial for immune defense.

Our study has some limitations. (a) The horizontal pleiotropy of some of the results could not be corrected, which may affect the reliability of the results. (b) Infectious diseases are affected by a variety of confounding factors in clinical situations, and the conclusion of Mendelian analysis is only based on the results of statistical analysis, which needs to be verified by further clinical studies. (c) Our definition of bacterial and viral infections relied on ICD codes from the FinnGen database. This approach is subject to inherent limitations, including diagnostic inaccuracy, coding errors, and variability in clinical practice. Future studies using more precise phenotyping, such as laboratory-confirmed infection cases, would be needed to validate our findings. (d) To minimize population stratification, we restricted our study to individuals of European ancestry. However, a limitation arises from using the FinnGen database for infection outcomes, as the Finnish population is a genetic isolate (47). Consequently, vitamin genetic instruments derived from the UK Biobank may not be fully transferable due to differences in linkage disequilibrium or allele frequencies. We employed a stringent LD clumping threshold (r² < 0.001) and multiple analytical methods to ensure robust findings. While these steps reduce the risk, residual stratification cannot be ruled out, necessitating future replication in genetically diverse populations.

## 5 Conclusion

Elevated circulating vitamin E levels may lead to an increased risk of viral infectious diseases, and bacterial infectious diseases may lead to a decreased circulating 25 (OH) D level.

## Data Availability

Publicly available datasets were analyzed in this study. This data can be found here: https://www.finngen.fi/en/access_results; https://www.ukbiobank.ac.uk/.
