# Supplementary material for: Causality of circulating vitamins on infectious diseases: integrating Mendelian randomization and in vivo evidence
Source: Front Immunol. 2025 Dec 1;16:1674678. doi: 10.3389/fimmu.2025.1674678 (PMC12702853; doi:10.3389/fimmu.2025.1674678)
Supplement: Supplementary file 1 [file Table1.docx]

| Exposure | Outcome | Mean F statistic | SNP | EA | OA | b_exp | se_exp | pval_exp | b_otcm | se_otcm | pval_otcm |  |
| --- | --- | --- | --- | --- | --- | --- | --- | --- | --- | --- | --- | --- |
| Vitamin A | Viral infection | 25.106 | rs10802946 | T | C | 0.073226 | 0.015446 | 2.13E-06 | 0.013509 | 0.014692 | 0.357828 |  |
|  |  |  | rs11187539 | A | G | -0.07758 | 0.015744 | 8.32E-07 | -0.0288 | 0.015151 | 0.057298 |  |
|  |  |  | rs115971394 | T | C | 0.276481 | 0.057893 | 1.79E-06 | 0.087838 | 0.100816 | 0.383609 |  |
|  |  |  | rs11604481 | C | A | -0.08214 | 0.017827 | 4.07E-06 | 0.007327 | 0.017504 | 0.675492 |  |
|  |  |  | rs11624628 | T | C | -0.27188 | 0.059039 | 4.12E-06 | -0.01873 | 0.086647 | 0.828879 |  |
|  |  |  | rs144434711 | C | G | 0.232473 | 0.048348 | 1.52E-06 | -0.01076 | 0.031388 | 0.731705 |  |
|  |  |  | rs1667226 | T | A | 0.101271 | 0.015461 | 5.74E-11 | -0.01036 | 0.014583 | 0.477518 |  |
|  |  |  | rs1795977 | C | G | -0.12805 | 0.027901 | 4.44E-06 | 0.001437 | 0.018092 | 0.936695 |  |
|  |  |  | rs1883711 | C | G | -0.27778 | 0.045435 | 9.73E-10 | 0.009554 | 0.03028 | 0.752376 |  |
|  |  |  | rs2505415 | C | G | 0.081289 | 0.016912 | 1.54E-06 | -0.00157 | 0.015687 | 0.920518 |  |
|  |  |  | rs3752802 | A | G | 0.081945 | 0.016972 | 1.38E-06 | -0.00849 | 0.018362 | 0.643963 |  |
|  |  |  | rs4665985 | C | A | 0.080474 | 0.017274 | 3.18E-06 | 0.006492 | 0.017173 | 0.705424 |  |
|  |  |  | rs6831628 | G | A | 0.098467 | 0.021092 | 3.03E-06 | 0.027091 | 0.019652 | 0.168043 |  |
| Vitamin B6 | Viral infection | 23.218 | rs10138490 | C | T | -0.0535 | 0.011414 | 2.77E-06 | -0.0082937 | 0.030372 | 0.7848 |  |
|  |  |  | rs12198456 | T | C | 0.091953 | 0.019723 | 3.13E-06 | -0.142981 | 0.083351 | 0.086268 |  |
|  |  |  | rs12226112 | T | G | 0.028341 | 0.005727 | 7.46E-07 | 0.00365693 | 0.014782 | 0.80461 |  |
|  |  |  | rs12412051 | C | G | 0.070864 | 0.014977 | 2.23E-06 | -0.0218683 | 0.035584 | 0.538844 |  |
|  |  |  | rs141933624 | A | G | -0.08973 | 0.019281 | 3.26E-06 | 0.0363904 | 0.060913 | 0.550232 |  |
|  |  |  | rs155599 | C | T | 0.034253 | 0.005985 | 1.04E-08 | -0.0125308 | 0.016209 | 0.439486 |  |
|  |  |  | rs183178622 | T | C | -0.09895 | 0.020684 | 1.72E-06 | 0.00665111 | 0.05562 | 0.904816 |  |
|  |  |  | rs188211816 | A | G | -0.07862 | 0.016281 | 1.37E-06 | -0.107195 | 0.0384 | 0.005246 |  |
|  |  |  | rs34938615 | G | A | -0.12202 | 0.026442 | 3.93E-06 | -0.151823 | 0.127672 | 0.234376 |  |
|  |  |  | rs3745438 | C | T | -0.07127 | 0.015597 | 4.89E-06 | 0.0170584 | 0.023553 | 0.468906 |  |
|  |  |  | rs3772928 | C | T | -0.02924 | 0.005534 | 1.26E-07 | -1.63E-05 | 0.014998 | 0.999131 |  |
|  |  |  | rs67450584 | T | C | 0.036705 | 0.007457 | 8.57E-07 | -0.0123797 | 0.022392 | 0.58035 |  |
|  |  |  | rs7205927 | C | A | -0.02579 | 0.005535 | 3.18E-06 | -0.0271993 | 0.014795 | 0.066002 |  |
|  |  |  | rs7292147 | C | G | -0.02584 | 0.005506 | 2.70E-06 | 0.00363405 | 0.014672 | 0.80438 |  |
|  |  |  | rs74640671 | T | C | -0.12718 | 0.02738 | 3.40E-06 | -0.0019319 | 0.101483 | 0.984812 |  |
|  |  |  | rs77806858 | C | T | -0.05051 | 0.01061 | 1.93E-06 | 0.00123817 | 0.031709 | 0.968852 |  |
|  |  |  | rs9560457 | T | C | 0.025504 | 0.00554 | 4.15E-06 | 0.0107544 | 0.014928 | 0.471255 |  |
| Vitamin B12 | Viral infection | 22.637 | rs10749205 | C | T | 0.031561 | 0.006837 | 3.91E-06 | -0.00336 | 0.016899 | 0.842477 |  |
|  |  |  | rs10924919 | T | C | -0.02863 | 0.005642 | 3.90E-07 | -0.0112 | 0.015063 | 0.457162 |  |
|  |  |  | rs112961770 | C | G | -0.08885 | 0.018432 | 1.43E-06 | 0.321385 | 0.340621 | 0.345411 |  |
|  |  |  | rs12776611 | A | G | -0.08781 | 0.018896 | 3.36E-06 | 0.011285 | 0.049994 | 0.821422 |  |
|  |  |  | rs148901823 | G | A | -0.04863 | 0.010052 | 1.31E-06 | -0.02416 | 0.025785 | 0.348672 |  |
|  |  |  | rs193228340 | C | T | 0.156303 | 0.034011 | 4.31E-06 | -0.03238 | 0.167114 | 0.846377 |  |
|  |  |  | rs388561 | C | T | 0.040271 | 0.008549 | 2.47E-06 | 0.048484 | 0.021514 | 0.024218 |  |
|  |  |  | rs61994378 | C | T | 0.093456 | 0.019774 | 2.29E-06 | 0.014429 | 0.044464 | 0.74555 |  |
|  |  |  | rs67568068 | C | T | -0.03174 | 0.006647 | 1.80E-06 | -0.01002 | 0.015999 | 0.531157 |  |
| Vitamin C | Viral infection | 23.208 | rs114598078 | T | C | 0.065578 | 0.013764 | 1.89E-06 | -0.03544 | 0.039855 | 0.373931 |  |
|  |  |  | rs11650824 | A | T | 0.079479 | 0.015882 | 5.61E-07 | 0.015185 | 0.027996 | 0.587537 |  |
|  |  |  | rs17482258 | T | C | 0.042829 | 0.00926 | 3.75E-06 | 0.02098 | 0.022999 | 0.361658 |  |
|  |  |  | rs1883993 | A | G | 0.044959 | 0.009353 | 1.53E-06 | -0.00843 | 0.017746 | 0.634746 |  |
|  |  |  | rs4238567 | C | T | 0.025307 | 0.005508 | 4.34E-06 | -0.00236 | 0.014683 | 0.872256 |  |
|  |  |  | rs4481190 | C | A | -0.03064 | 0.005744 | 9.61E-08 | -0.01639 | 0.015149 | 0.27937 |  |
|  |  |  | rs61868302 | T | C | -0.0571 | 0.011839 | 1.41E-06 | -0.04707 | 0.038394 | 0.220216 |  |
|  |  |  | rs74978963 | T | C | 0.150814 | 0.031021 | 1.16E-06 | 0.013209 | 0.045222 | 0.770222 |  |
|  |  |  | rs7626478 | A | G | 0.02798 | 0.006101 | 4.52E-06 | -0.02662 | 0.016815 | 0.113456 |  |
|  |  |  | rs9540734 | A | G | -0.02593 | 0.005485 | 2.27E-06 | -0.00371 | 0.014582 | 0.799349 |  |
| Vitamin D | Viral infection | 21.956 | rs10469075 | T | C | -0.03246 | 0.007013 | 3.68E-06 | 0.006434 | 0.019975 | 0.747355 |  |
|  |  |  | rs117693112 | A | G | 0.068243 | 0.014479 | 2.44E-06 | -0.04178 | 0.054203 | 0.440827 |  |
|  |  |  | rs17301981 | C | T | -0.04379 | 0.009585 | 4.92E-06 | 0.023547 | 0.025897 | 0.363229 |  |
|  |  |  | rs2673883 | C | T | -0.03429 | 0.007054 | 1.17E-06 | 0.005088 | 0.019774 | 0.796949 |  |
|  |  |  | rs35775421 | A | G | -0.05597 | 0.012219 | 4.63E-06 | -0.01691 | 0.024803 | 0.495309 |  |
|  |  |  | rs57038272 | T | C | 0.033332 | 0.007237 | 4.11E-06 | -0.0392 | 0.017817 | 0.027807 |  |
|  |  |  | rs582962 | A | G | -0.02832 | 0.006081 | 3.21E-06 | 0.02074 | 0.014961 | 0.165672 |  |
|  |  |  | rs61942184 | C | G | 0.082217 | 0.017757 | 3.66E-06 | -0.09103 | 0.053065 | 0.086264 |  |
|  |  |  | rs679830 | C | T | -0.06018 | 0.012755 | 2.38E-06 | -0.01162 | 0.031505 | 0.7123 |  |
|  |  |  | rs74593039 | C | G | 0.035005 | 0.007539 | 3.43E-06 | -0.00262 | 0.02051 | 0.898305 |  |
|  |  |  | rs75713989 | T | C | 0.039147 | 0.008167 | 1.64E-06 | 0.006904 | 0.022844 | 0.762483 |  |
|  |  |  | rs80261862 | T | C | -0.04487 | 0.009267 | 1.28E-06 | -0.00814 | 0.01885 | 0.665673 |  |
|  |  |  | rs9328367 | T | A | 0.02618 | 0.005617 | 3.15E-06 | -0.0058 | 0.014662 | 0.692637 |  |
| 25(OH)D | Viral infection | 21.956 | rs10070734 | T | C | -0.01083 | 0.002237 | 1.29E-06 | -0.0156867 | 0.016414 | 0.339222 |  |
|  |  |  | rs10090648 | A | G | -0.01488 | 0.00312 | 1.86E-06 | -0.028757 | 0.025135 | 0.252588 |  |
|  |  |  | rs10121187 | G | C | 0.00931 | 0.002036 | 4.81E-06 | 0.0394093 | 0.014586 | 0.006896 |  |
|  |  |  | rs10171594 | G | A | 0.01376 | 0.002705 | 3.62E-07 | -0.00716134 | 0.016396 | 0.662274 |  |
|  |  |  | rs10277163 | G | A | -0.01433 | 0.002351 | 1.08E-09 | 0.0111816 | 0.01739 | 0.520238 |  |
|  |  |  | rs1038165 | C | T | -0.01151 | 0.002056 | 2.15E-08 | 0.0188789 | 0.014723 | 0.199743 |  |
|  |  |  | rs1042034 | C | T | 0.015125 | 0.0025 | 1.45E-09 | 0.015961 | 0.016388 | 0.330079 |  |
|  |  |  | rs10438978 | T | C | 0.017224 | 0.002644 | 7.34E-11 | -0.0258436 | 0.018973 | 0.173167 |  |
|  |  |  | rs1047891 | A | C | -0.0134 | 0.00218 | 7.96E-10 | 0.00335124 | 0.015598 | 0.829882 |  |
|  |  |  | rs1048328 | A | G | 0.03135 | 0.003744 | 5.58E-17 | 0.00677112 | 0.024303 | 0.780543 |  |
|  |  |  | rs10521222 | T | C | 0.025151 | 0.004857 | 2.24E-07 | 0.0024753 | 0.046396 | 0.957452 |  |
|  |  |  | rs10822184 | C | T | -0.00992 | 0.002029 | 1.01E-06 | -0.0193919 | 0.014617 | 0.184623 |  |
|  |  |  | rs10859995 | T | C | 0.043626 | 0.002055 | 4.60E-100 | 0.0184445 | 0.015391 | 0.230769 |  |
|  |  |  | rs10896045 | A | G | -0.01278 | 0.002229 | 9.81E-09 | 0.00585188 | 0.016322 | 0.719944 |  |
|  |  |  | rs10952882 | A | G | 0.0098 | 0.002042 | 1.58E-06 | -0.00994483 | 0.01467 | 0.497817 |  |
|  |  |  | rs11060406 | T | C | -0.03022 | 0.005581 | 6.10E-08 | 0.0527216 | 0.033913 | 0.120038 |  |
|  |  |  | rs112072036 | G | A | -0.20594 | 0.004981 | 0 | -0.0468357 | 0.038517 | 0.223995 |  |
|  |  |  | rs11207969 | G | A | 0.02094 | 0.002127 | 7.14E-23 | 0.000677962 | 0.016502 | 0.967229 |  |
|  |  |  | rs11264361 | G | T | 0.017488 | 0.002341 | 7.97E-14 | -0.0183975 | 0.016252 | 0.25763 |  |
|  |  |  | rs1128535 | C | T | -0.01641 | 0.002029 | 6.06E-16 | -0.00303971 | 0.015175 | 0.841242 |  |
|  |  |  | rs115288876 | A | G | 0.078806 | 0.004982 | 2.36E-56 | -0.0771088 | 0.037465 | 0.039573 |  |
|  |  |  | rs11542462 | A | G | -0.02478 | 0.002983 | 9.72E-17 | 0.0114847 | 0.025045 | 0.646553 |  |
|  |  |  | rs11625899 | T | C | 0.009892 | 0.002124 | 3.21E-06 | -0.0049054 | 0.015082 | 0.744996 |  |
|  |  |  | rs11637664 | T | A | -0.01033 | 0.002036 | 3.92E-07 | 0.00925143 | 0.014586 | 0.5259 |  |
|  |  |  | rs11651767 | A | G | 0.009827 | 0.002141 | 4.42E-06 | 0.0171763 | 0.015033 | 0.253207 |  |
|  |  |  | rs11745926 | C | T | -0.01073 | 0.002339 | 4.48E-06 | 0.00657416 | 0.015849 | 0.678289 |  |
|  |  |  | rs11752007 | C | T | 0.016456 | 0.003074 | 8.61E-08 | 0.0219256 | 0.023398 | 0.34871 |  |
|  |  |  | rs117862422 | C | T | -0.05046 | 0.008655 | 5.52E-09 | -0.00936762 | 0.036319 | 0.796462 |  |
|  |  |  | rs11791258 | A | G | 0.014081 | 0.002581 | 4.85E-08 | 0.018755 | 0.021771 | 0.388981 |  |
|  |  |  | rs11865670 | G | A | 0.010082 | 0.002138 | 2.42E-06 | 0.0228919 | 0.014586 | 0.116546 |  |
|  |  |  | rs11867297 | T | C | 0.013543 | 0.002095 | 1.01E-10 | 0.0225043 | 0.015358 | 0.142835 |  |
|  |  |  | rs11898833 | C | T | 0.011351 | 0.002344 | 1.28E-06 | -0.0269438 | 0.017155 | 0.116261 |  |
|  |  |  | rs12056768 | T | G | 0.023196 | 0.002064 | 2.65E-29 | 0.0123191 | 0.014562 | 0.397551 |  |
|  |  |  | rs12147536 | C | T | -0.01354 | 0.002822 | 1.61E-06 | 0.0215321 | 0.02018 | 0.285968 |  |
|  |  |  | rs12369406 | T | C | 0.009818 | 0.002098 | 2.88E-06 | 0.0126572 | 0.015298 | 0.408028 |  |
|  |  |  | rs12403824 | G | C | 0.010675 | 0.002221 | 1.54E-06 | 0.00900657 | 0.015412 | 0.558965 |  |
|  |  |  | rs12462826 | A | G | -0.01321 | 0.002115 | 4.18E-10 | -0.00811773 | 0.015469 | 0.59974 |  |
|  |  |  | rs1260326 | T | C | -0.01972 | 0.002074 | 1.96E-21 | 0.00869804 | 0.015273 | 0.569002 |  |
|  |  |  | rs12616170 | C | A | -0.00999 | 0.002174 | 4.32E-06 | 0.02115 | 0.015321 | 0.167449 |  |
|  |  |  | rs12713125 | G | T | -0.01122 | 0.002401 | 2.94E-06 | -0.00426087 | 0.018059 | 0.81348 |  |
|  |  |  | rs12775091 | T | C | 0.015562 | 0.002477 | 3.33E-10 | -0.001419 | 0.01602 | 0.929418 |  |
|  |  |  | rs12816349 | A | G | 0.011015 | 0.002372 | 3.42E-06 | 0.00354249 | 0.018781 | 0.850387 |  |
|  |  |  | rs12919213 | T | C | -0.01495 | 0.002875 | 1.99E-07 | -0.0295197 | 0.022626 | 0.192001 |  |
|  |  |  | rs12949853 | G | A | -0.01424 | 0.002616 | 5.24E-08 | 0.00845674 | 0.019205 | 0.659683 |  |
|  |  |  | rs13064022 | A | G | 0.019893 | 0.004222 | 2.46E-06 | -0.0313437 | 0.025505 | 0.219102 |  |
|  |  |  | rs13076508 | C | T | 0.025054 | 0.00451 | 2.78E-08 | -0.0152917 | 0.03671 | 0.677004 |  |
|  |  |  | rs13079205 | G | A | 0.011368 | 0.0023 | 7.71E-07 | 0.0144789 | 0.017812 | 0.4163 |  |
|  |  |  | rs13201062 | A | C | 0.010002 | 0.002073 | 1.40E-06 | 0.0255873 | 0.014597 | 0.079614 |  |
|  |  |  | rs1321247 | T | A | -0.02218 | 0.003366 | 4.36E-11 | -3.84E-05 | 0.022994 | 0.998667 |  |
|  |  |  | rs1324190 | T | C | 0.010232 | 0.002099 | 1.09E-06 | -0.0188774 | 0.014737 | 0.200214 |  |
|  |  |  | rs13278404 | C | G | -0.01416 | 0.002942 | 1.48E-06 | -0.0090208 | 0.026705 | 0.735521 |  |
|  |  |  | rs13294734 | T | C | 0.012568 | 0.002058 | 1.02E-09 | -0.0176021 | 0.014568 | 0.226951 |  |
|  |  |  | rs13389628 | T | C | 0.02011 | 0.003934 | 3.20E-07 | -0.0286912 | 0.027917 | 0.304071 |  |
|  |  |  | rs1343776 | A | G | 0.018076 | 0.00245 | 1.62E-13 | 0.00782428 | 0.017625 | 0.657092 |  |
|  |  |  | rs1369136 | A | G | -0.02216 | 0.004615 | 1.58E-06 | -0.0464192 | 0.030232 | 0.124674 |  |
|  |  |  | rs138335 | C | G | 0.013766 | 0.002151 | 1.56E-10 | 0.00767745 | 0.015049 | 0.609943 |  |
|  |  |  | rs1384687 | A | G | -0.01687 | 0.002997 | 1.82E-08 | -0.00177395 | 0.016533 | 0.914553 |  |
|  |  |  | rs1395975 | A | G | 0.00994 | 0.002053 | 1.28E-06 | 0.0217553 | 0.014687 | 0.138524 |  |
|  |  |  | rs141335934 | T | C | 0.01837 | 0.003943 | 3.18E-06 | 0.00682015 | 0.024491 | 0.780649 |  |
|  |  |  | rs142004400 | C | A | -0.031 | 0.005596 | 3.01E-08 | 0.0234965 | 0.051889 | 0.650678 |  |
|  |  |  | rs142158911 | A | G | 0.026284 | 0.003234 | 4.43E-16 | 0.017789 | 0.024027 | 0.459067 |  |
|  |  |  | rs1432902 | A | C | -0.02036 | 0.004251 | 1.67E-06 | 0.0120618 | 0.029871 | 0.686366 |  |
|  |  |  | rs1466414 | G | A | 0.011243 | 0.002247 | 5.65E-07 | -0.021435 | 0.016214 | 0.186159 |  |
|  |  |  | rs1532085 | A | G | -0.02528 | 0.002086 | 8.60E-34 | 0.000953832 | 0.014727 | 0.94836 |  |
|  |  |  | rs1548904 | A | C | -0.00997 | 0.002076 | 1.56E-06 | 0.0169495 | 0.014757 | 0.250729 |  |
|  |  |  | rs1558902 | A | T | 0.011521 | 0.002066 | 2.48E-08 | -0.0106149 | 0.014717 | 0.470732 |  |
|  |  |  | rs1664781 | G | A | 0.010737 | 0.0022 | 1.06E-06 | 0.00282527 | 0.015443 | 0.854836 |  |
|  |  |  | rs1684600 | T | C | -0.01253 | 0.002217 | 1.59E-08 | -0.0137523 | 0.016762 | 0.411965 |  |
|  |  |  | rs16848096 | G | T | 0.017428 | 0.003571 | 1.06E-06 | 0.00922043 | 0.022498 | 0.681924 |  |
|  |  |  | rs16879122 | G | A | 0.017407 | 0.003812 | 4.96E-06 | 0.00952973 | 0.023709 | 0.687717 |  |
|  |  |  | rs17057661 | C | T | 0.010927 | 0.002146 | 3.57E-07 | 0.0118066 | 0.015675 | 0.451316 |  |
|  |  |  | rs17105586 | G | T | 0.009942 | 0.002112 | 2.52E-06 | 0.00349746 | 0.014941 | 0.814921 |  |
|  |  |  | rs1714336 | T | C | 0.014427 | 0.002082 | 4.23E-12 | 0.0191153 | 0.014965 | 0.201496 |  |
|  |  |  | rs17207784 | C | T | -0.01349 | 0.002171 | 5.14E-10 | 0.00707597 | 0.015667 | 0.651528 |  |
|  |  |  | rs17216177 | C | T | 0.02044 | 0.004368 | 2.88E-06 | -0.0683793 | 0.046689 | 0.143041 |  |
|  |  |  | rs17593743 | C | A | 0.015751 | 0.003252 | 1.28E-06 | -0.0148569 | 0.028654 | 0.604116 |  |
|  |  |  | rs17688247 | T | C | -0.01325 | 0.002872 | 3.95E-06 | -0.00788639 | 0.017729 | 0.65644 |  |
|  |  |  | rs1792287 | G | A | 0.023152 | 0.002307 | 1.06E-23 | -0.00966406 | 0.016037 | 0.546755 |  |
|  |  |  | rs1800588 | T | C | -0.0305 | 0.002469 | 4.73E-35 | 0.00207521 | 0.016819 | 0.9018 |  |
|  |  |  | rs1800775 | A | C | -0.01742 | 0.002029 | 9.23E-18 | 0.0123073 | 0.014513 | 0.396424 |  |
|  |  |  | rs1858889 | A | C | -0.01345 | 0.002031 | 3.49E-11 | -0.00701547 | 0.014588 | 0.630588 |  |
|  |  |  | rs1871395 | G | A | -0.02037 | 0.002827 | 5.72E-13 | -0.00400269 | 0.016271 | 0.805685 |  |
|  |  |  | rs1894100 | T | G | -0.10215 | 0.002592 | 0 | 0.0159308 | 0.015234 | 0.295683 |  |
|  |  |  | rs1949633 | T | C | -0.01142 | 0.002086 | 4.45E-08 | -0.0223477 | 0.01522 | 0.142006 |  |
|  |  |  | rs1993116 | A | G | 0.084686 | 0.00206 | 0 | 0.00648059 | 0.014665 | 0.658544 |  |
|  |  |  | rs2069502 | T | C | -0.01166 | 0.002172 | 7.93E-08 | -0.0174811 | 0.015341 | 0.254477 |  |
|  |  |  | rs2070737 | T | A | -0.01062 | 0.002112 | 4.89E-07 | 0.0221125 | 0.015957 | 0.165818 |  |
|  |  |  | rs2074735 | C | G | 0.029267 | 0.00412 | 1.22E-12 | 0.000965819 | 0.020906 | 0.963152 |  |
|  |  |  | rs2157829 | A | G | 0.009993 | 0.002166 | 3.94E-06 | 0.016947 | 0.016776 | 0.312401 |  |
|  |  |  | rs2165331 | G | T | -0.01009 | 0.002207 | 4.79E-06 | 0.00872955 | 0.016032 | 0.586081 |  |
|  |  |  | rs2229742 | C | G | -0.02498 | 0.003314 | 4.75E-14 | 0.00310967 | 0.023182 | 0.893293 |  |
|  |  |  | rs2239537 | T | G | -0.01242 | 0.002368 | 1.55E-07 | 0.0189926 | 0.017632 | 0.281398 |  |
|  |  |  | rs2245133 | C | T | -0.02129 | 0.00274 | 7.80E-15 | 0.00124848 | 0.017765 | 0.943972 |  |
|  |  |  | rs2297991 | T | C | -0.01275 | 0.002256 | 1.57E-08 | -0.0180547 | 0.01537 | 0.24012 |  |
|  |  |  | rs2336865 | C | T | 0.010225 | 0.002111 | 1.28E-06 | 0.0258357 | 0.015393 | 0.093263 |  |
|  |  |  | rs2470937 | A | T | -0.01105 | 0.002042 | 6.30E-08 | 0.00492207 | 0.014703 | 0.737804 |  |
|  |  |  | rs2472466 | A | G | -0.00972 | 0.002068 | 2.59E-06 | -0.0109467 | 0.014698 | 0.456397 |  |
|  |  |  | rs2494429 | A | G | 0.014846 | 0.002673 | 2.80E-08 | -0.00417428 | 0.019144 | 0.827391 |  |
|  |  |  | rs2595644 | T | G | -0.01226 | 0.002097 | 4.97E-09 | 0.00360821 | 0.015352 | 0.814187 |  |
|  |  |  | rs2710651 | G | A | 0.011589 | 0.002035 | 1.23E-08 | 0.0102489 | 0.014592 | 0.482465 |  |
|  |  |  | rs2756119 | A | G | 0.012143 | 0.00211 | 8.71E-09 | -0.0131984 | 0.01501 | 0.379229 |  |
|  |  |  | rs2784773 | T | C | 0.010925 | 0.002129 | 2.87E-07 | 0.00706249 | 0.015825 | 0.655398 |  |
|  |  |  | rs2807834 | T | G | 0.015063 | 0.002187 | 5.66E-12 | -0.0118976 | 0.016254 | 0.464175 |  |
|  |  |  | rs28435470 | G | A | 0.01187 | 0.002148 | 3.29E-08 | 0.00558607 | 0.014793 | 0.705708 |  |
|  |  |  | rs2847500 | A | G | -0.02255 | 0.003087 | 2.77E-13 | 0.0260207 | 0.020684 | 0.208387 |  |
|  |  |  | rs290400 | G | A | 0.013097 | 0.002163 | 1.41E-09 | 0.00608275 | 0.014985 | 0.684789 |  |
|  |  |  | rs2977273 | G | A | -0.0115 | 0.002241 | 2.84E-07 | -0.0181149 | 0.015897 | 0.254477 |  |
|  |  |  | rs3114045 | T | C | 0.022174 | 0.00298 | 1.00E-13 | 0.0155159 | 0.021913 | 0.478892 |  |
|  |  |  | rs317195 | C | T | -0.01437 | 0.003103 | 3.66E-06 | -0.0153838 | 0.016973 | 0.364726 |  |
|  |  |  | rs34177108 | A | C | -0.0115 | 0.002321 | 7.28E-07 | -0.00566178 | 0.018269 | 0.75662 |  |
|  |  |  | rs34186890 | G | A | -0.01569 | 0.002318 | 1.33E-11 | -0.0114132 | 0.016926 | 0.500129 |  |
|  |  |  | rs34726834 | T | C | 0.014013 | 0.002349 | 2.42E-09 | 0.0129461 | 0.01593 | 0.416403 |  |
|  |  |  | rs35270497 | T | C | 0.015672 | 0.002681 | 5.08E-09 | -0.0144888 | 0.017874 | 0.417594 |  |
|  |  |  | rs35285316 | T | C | 0.014437 | 0.002706 | 9.56E-08 | -0.021845 | 0.022119 | 0.323349 |  |
|  |  |  | rs35634730 | T | C | 0.014988 | 0.002993 | 5.52E-07 | -0.0266259 | 0.027728 | 0.336936 |  |
|  |  |  | rs35947684 | C | G | 0.010118 | 0.00207 | 1.02E-06 | -0.0105937 | 0.014585 | 0.467623 |  |
|  |  |  | rs3732220 | A | G | -0.04784 | 0.003633 | 1.31E-39 | -0.00268833 | 0.027695 | 0.922672 |  |
|  |  |  | rs3750569 | G | A | 0.013667 | 0.002657 | 2.68E-07 | 0.0180354 | 0.020051 | 0.368395 |  |
|  |  |  | rs3768790 | A | G | -0.02032 | 0.003867 | 1.49E-07 | 0.00587188 | 0.0246 | 0.811346 |  |
|  |  |  | rs3784441 | A | G | -0.01297 | 0.00244 | 1.06E-07 | 0.00870511 | 0.016305 | 0.593421 |  |
|  |  |  | rs3890624 | G | A | 0.010535 | 0.002108 | 5.81E-07 | 0.0107598 | 0.015348 | 0.483259 |  |
|  |  |  | rs3924150 | C | T | -0.01126 | 0.002063 | 4.82E-08 | -0.00535853 | 0.014697 | 0.715415 |  |
|  |  |  | rs4364259 | A | G | 0.017241 | 0.002567 | 1.86E-11 | -0.00356182 | 0.017652 | 0.840085 |  |
|  |  |  | rs4420638 | G | A | -0.0193 | 0.002659 | 3.95E-13 | -0.0282444 | 0.016356 | 0.0842 |  |
|  |  |  | rs4441458 | T | C | -0.01069 | 0.002262 | 2.28E-06 | -0.00827023 | 0.015755 | 0.599625 |  |
|  |  |  | rs4504191 | T | G | -0.01088 | 0.002341 | 3.33E-06 | 0.0145293 | 0.017416 | 0.404135 |  |
|  |  |  | rs4580037 | C | A | -0.01356 | 0.002251 | 1.68E-09 | -0.0125073 | 0.01642 | 0.44623 |  |
|  |  |  | rs4678803 | A | G | 0.012135 | 0.00226 | 7.87E-08 | 0.012818 | 0.016998 | 0.450796 |  |
|  |  |  | rs4694423 | A | C | -0.0937 | 0.002056 | 0 | 0.0292016 | 0.01471 | 0.047124 |  |
|  |  |  | rs4715218 | G | A | 0.010448 | 0.002222 | 2.57E-06 | -0.00746842 | 0.015838 | 0.637238 |  |
|  |  |  | rs4849777 | C | A | -0.01253 | 0.00252 | 6.69E-07 | 0.0259875 | 0.017806 | 0.144439 |  |
|  |  |  | rs4916928 | C | A | 0.013428 | 0.0029 | 3.65E-06 | 0.0095551 | 0.019999 | 0.632807 |  |
|  |  |  | rs4924 | C | T | -0.0104 | 0.002036 | 3.21E-07 | 0.00929604 | 0.014788 | 0.52959 |  |
|  |  |  | rs4983329 | C | A | -0.00941 | 0.002046 | 4.22E-06 | -0.00266605 | 0.014613 | 0.855238 |  |
|  |  |  | rs5011717 | G | A | 0.009609 | 0.002044 | 2.59E-06 | 0.000317716 | 0.014668 | 0.982719 |  |
|  |  |  | rs512083 | C | T | 0.012217 | 0.002043 | 2.23E-09 | 0.00744722 | 0.014553 | 0.608834 |  |
|  |  |  | rs55853471 | G | C | -0.05022 | 0.005089 | 5.72E-23 | 0.0150198 | 0.038126 | 0.693613 |  |
|  |  |  | rs57601828 | T | A | 0.011542 | 0.002082 | 2.96E-08 | -0.0144492 | 0.014595 | 0.322157 |  |
|  |  |  | rs5770794 | T | C | -0.01331 | 0.002211 | 1.74E-09 | 0.0229861 | 0.015083 | 0.127508 |  |
|  |  |  | rs59488684 | C | A | 0.01003 | 0.002057 | 1.09E-06 | -0.0168386 | 0.014571 | 0.247818 |  |
|  |  |  | rs6001026 | T | C | 0.01039 | 0.002148 | 1.32E-06 | -0.00053383 | 0.015361 | 0.972277 |  |
|  |  |  | rs6003465 | C | T | -0.011 | 0.002164 | 3.74E-07 | -0.00838135 | 0.016375 | 0.608768 |  |
|  |  |  | rs6129648 | G | A | 0.014063 | 0.002106 | 2.44E-11 | -0.00498405 | 0.015455 | 0.747082 |  |
|  |  |  | rs61698755 | T | C | 0.011465 | 0.002051 | 2.25E-08 | 0.021501 | 0.014676 | 0.142894 |  |
|  |  |  | rs61747728 | T | C | 0.030306 | 0.005269 | 8.83E-09 | 0.0239054 | 0.030259 | 0.429511 |  |
|  |  |  | rs61813875 | G | C | 0.082129 | 0.006589 | 1.16E-35 | -0.0208735 | 0.081241 | 0.797231 |  |
|  |  |  | rs62007299 | G | A | 0.012421 | 0.002244 | 3.12E-08 | -0.0011355 | 0.015546 | 0.941773 |  |
|  |  |  | rs62012766 | C | T | -0.01613 | 0.002777 | 6.32E-09 | 0.0298461 | 0.017278 | 0.084085 |  |
|  |  |  | rs62166770 | G | T | 0.011049 | 0.002265 | 1.07E-06 | 0.0104646 | 0.018302 | 0.56747 |  |
|  |  |  | rs62319568 | G | A | 0.014434 | 0.002851 | 4.13E-07 | -4.64E-05 | 0.01895 | 0.998044 |  |
|  |  |  | rs6438900 | G | C | 0.015049 | 0.00234 | 1.27E-10 | 0.0181029 | 0.015843 | 0.253181 |  |
|  |  |  | rs6599618 | T | C | -0.01005 | 0.002086 | 1.46E-06 | -0.0134799 | 0.014622 | 0.356594 |  |
|  |  |  | rs6600876 | G | T | -0.03124 | 0.002036 | 3.94E-53 | -0.00072328 | 0.014666 | 0.960665 |  |
|  |  |  | rs6706116 | T | C | 0.010184 | 0.002077 | 9.48E-07 | -0.0267962 | 0.014942 | 0.072907 |  |
|  |  |  | rs6775277 | A | G | 0.010629 | 0.002194 | 1.27E-06 | 0.0126179 | 0.015189 | 0.406128 |  |
|  |  |  | rs6834488 | T | C | -0.01445 | 0.002059 | 2.26E-12 | -0.00076112 | 0.015533 | 0.960919 |  |
|  |  |  | rs6871090 | A | C | 0.010072 | 0.002148 | 2.74E-06 | -0.0126851 | 0.014679 | 0.387496 |  |
|  |  |  | rs6877237 | G | C | -0.01161 | 0.002378 | 1.04E-06 | -0.0432142 | 0.016338 | 0.008169 |  |
|  |  |  | rs6963 | A | T | -0.01206 | 0.002253 | 8.66E-08 | -0.0085092 | 0.016979 | 0.616247 |  |
|  |  |  | rs6972278 | T | G | 0.009663 | 0.002104 | 4.36E-06 | 0.0271237 | 0.014956 | 0.069745 |  |
|  |  |  | rs6982486 | G | A | -0.01001 | 0.002151 | 3.28E-06 | 0.00438864 | 0.01495 | 0.769104 |  |
|  |  |  | rs703491 | T | C | 0.012157 | 0.002573 | 2.31E-06 | 0.0298303 | 0.019201 | 0.12029 |  |
|  |  |  | rs7077708 | G | A | -0.01084 | 0.002211 | 9.54E-07 | 0.0215352 | 0.0146 | 0.140206 |  |
|  |  |  | rs71601787 | A | G | 0.044836 | 0.002178 | 3.97E-94 | 0.0009826 | 0.01595 | 0.950876 |  |
|  |  |  | rs7235662 | A | G | 0.013349 | 0.002911 | 4.53E-06 | 0.0146744 | 0.020767 | 0.47981 |  |
|  |  |  | rs727857 | G | A | 0.012055 | 0.002099 | 9.27E-09 | -0.0187652 | 0.014618 | 0.199231 |  |
|  |  |  | rs72997688 | G | A | 0.025696 | 0.004374 | 4.24E-09 | 0.0375969 | 0.028519 | 0.1874 |  |
|  |  |  | rs73413596 | C | T | 0.022347 | 0.003889 | 9.15E-09 | 0.0574111 | 0.031162 | 0.065428 |  |
|  |  |  | rs740772 | A | G | 0.009806 | 0.002102 | 3.08E-06 | 0.0150854 | 0.014786 | 0.307615 |  |
|  |  |  | rs742493 | C | T | 0.018353 | 0.003206 | 1.04E-08 | 0.0267087 | 0.024739 | 0.280308 |  |
|  |  |  | rs745570 | A | G | 0.010469 | 0.002031 | 2.53E-07 | 0.0252217 | 0.01453 | 0.082591 |  |
|  |  |  | rs7528419 | G | A | 0.021539 | 0.002432 | 8.17E-19 | -0.0136498 | 0.017728 | 0.441313 |  |
|  |  |  | rs7569755 | A | G | 0.013639 | 0.002256 | 1.49E-09 | 0.00992636 | 0.017754 | 0.576078 |  |
|  |  |  | rs7580771 | T | G | -0.01656 | 0.002665 | 5.15E-10 | 0.0221825 | 0.017513 | 0.20528 |  |
|  |  |  | rs75936148 | A | G | -0.01765 | 0.003532 | 5.76E-07 | 0.0401668 | 0.028116 | 0.153116 |  |
|  |  |  | rs7652808 | T | G | 0.021286 | 0.002126 | 1.36E-23 | -0.012733 | 0.016327 | 0.435452 |  |
|  |  |  | rs7657132 | G | A | -0.0136 | 0.002206 | 6.99E-10 | 0.0130606 | 0.015706 | 0.40564 |  |
|  |  |  | rs7712001 | G | T | 0.011939 | 0.002062 | 7.05E-09 | 0.00294038 | 0.015 | 0.844587 |  |
|  |  |  | rs77521820 | T | G | 0.020809 | 0.003989 | 1.82E-07 | -0.018243 | 0.027615 | 0.508854 |  |
|  |  |  | rs77532868 | T | C | 0.025956 | 0.004563 | 1.28E-08 | 0.0246587 | 0.041403 | 0.551456 |  |
|  |  |  | rs77559960 | T | G | -0.0131 | 0.002535 | 2.39E-07 | -0.00332292 | 0.01854 | 0.857758 |  |
|  |  |  | rs7780345 | C | T | -0.01723 | 0.003609 | 1.81E-06 | 0.0210311 | 0.027438 | 0.443385 |  |
|  |  |  | rs77924615 | A | G | -0.01525 | 0.00259 | 3.94E-09 | 0.00513174 | 0.017525 | 0.769655 |  |
|  |  |  | rs77960347 | G | A | -0.05257 | 0.00906 | 6.53E-09 | 0.0309639 | 0.084889 | 0.715293 |  |
|  |  |  | rs78649910 | A | T | -0.01914 | 0.003322 | 8.33E-09 | 0.0361806 | 0.022311 | 0.104877 |  |
|  |  |  | rs794000 | A | G | 0.018672 | 0.00359 | 1.98E-07 | -0.0375709 | 0.027712 | 0.175167 |  |
|  |  |  | rs7955128 | A | T | -0.01306 | 0.002039 | 1.48E-10 | 0.000542264 | 0.014684 | 0.970542 |  |
|  |  |  | rs79687284 | C | G | -0.02721 | 0.005543 | 9.19E-07 | 0.0205268 | 0.05556 | 0.711787 |  |
|  |  |  | rs79699939 | C | A | 0.0248 | 0.004392 | 1.64E-08 | 0.0305042 | 0.02893 | 0.2917 |  |
|  |  |  | rs7997035 | T | C | -0.01037 | 0.002102 | 8.11E-07 | 0.0278152 | 0.015241 | 0.067989 |  |
|  |  |  | rs8018720 | G | C | 0.034496 | 0.002661 | 1.93E-38 | -0.0276008 | 0.020426 | 0.176622 |  |
|  |  |  | rs8026614 | T | C | -0.0097 | 0.002044 | 2.08E-06 | -0.0144497 | 0.014569 | 0.321271 |  |
|  |  |  | rs804281 | A | G | -0.0159 | 0.00206 | 1.20E-14 | -0.0158085 | 0.01577 | 0.316119 |  |
|  |  |  | rs8063565 | G | C | -0.0115 | 0.002296 | 5.42E-07 | -0.00015191 | 0.016 | 0.992425 |  |
|  |  |  | rs8093467 | T | C | 0.009886 | 0.002074 | 1.86E-06 | 0.0116386 | 0.0148 | 0.43163 |  |
|  |  |  | rs8107974 | T | A | 0.035567 | 0.003823 | 1.36E-20 | 0.0277079 | 0.029641 | 0.349893 |  |
|  |  |  | rs8121940 | G | C | -0.04356 | 0.002549 | 1.77E-65 | -0.0326259 | 0.017209 | 0.057983 |  |
|  |  |  | rs818168 | C | T | 0.010466 | 0.002151 | 1.14E-06 | -0.00981801 | 0.014672 | 0.503381 |  |
|  |  |  | rs8614 | A | C | -0.0143 | 0.002629 | 5.32E-08 | -0.00647585 | 0.020173 | 0.748192 |  |
|  |  |  | rs9363415 | T | C | 0.010915 | 0.002033 | 7.93E-08 | 0.027451 | 0.014617 | 0.060384 |  |
|  |  |  | rs9375037 | C | A | 0.011706 | 0.002054 | 1.21E-08 | -0.00926412 | 0.014588 | 0.5254 |  |
|  |  |  | rs9559508 | T | C | -0.01348 | 0.002616 | 2.54E-07 | -0.0001295 | 0.01808 | 0.994285 |  |
|  |  |  | rs964184 | G | C | -0.04068 | 0.002989 | 3.50E-42 | -0.0223901 | 0.020682 | 0.278993 |  |
|  |  |  | rs986649 | G | A | 0.012864 | 0.002178 | 3.51E-09 | -0.0131548 | 0.014997 | 0.380407 |  |
|  |  |  | rs9892194 | A | G | 0.010602 | 0.002062 | 2.71E-07 | 0.00526965 | 0.014863 | 0.722923 |  |
|  |  |  | rs9946771 | T | C | -0.0234 | 0.004077 | 9.47E-09 | -0.0366284 | 0.027024 | 0.175294 |  |
| Vitamin E | Viral infection | 23.091 | rs111306778 | A | G | -0.04797 | 0.009572 | 5.42E-07 | 0.000508 | 0.023929 | 0.983055 |  |
|  |  |  | rs12421920 | G | A | -0.04345 | 0.009385 | 3.67E-06 | -0.0384 | 0.033288 | 0.248712 |  |
|  |  |  | rs12899673 | A | G | 0.026895 | 0.005818 | 3.79E-06 | -0.00604 | 0.015151 | 0.689951 |  |
|  |  |  | rs2723979 | G | T | -0.02661 | 0.005527 | 1.47E-06 | -0.02431 | 0.014678 | 0.097717 |  |
|  |  |  | rs35218694 | G | A | -0.07416 | 0.01531 | 1.27E-06 | 0.017391 | 0.051462 | 0.735402 |  |
|  |  |  | rs4903544 | T | C | -0.02951 | 0.006021 | 9.53E-07 | -0.01364 | 0.015491 | 0.378463 |  |
|  |  |  | rs536912 | A | C | 0.03046 | 0.006201 | 9.01E-07 | 0.003009 | 0.016672 | 0.856781 |  |
|  |  |  | rs6033 | G | A | -0.05166 | 0.010556 | 9.91E-07 | -0.04843 | 0.023041 | 0.035576 |  |
|  |  |  | rs71385328 | G | A | 0.130015 | 0.026206 | 7.01E-07 | 0.059341 | 0.031195 | 0.057139 |  |
|  |  |  | rs715455 | A | C | -0.03984 | 0.008604 | 3.65E-06 | -0.02624 | 0.022043 | 0.233827 |  |
|  |  |  | rs79966958 | T | C | -0.11653 | 0.024505 | 1.98E-06 | 0.018294 | 0.054379 | 0.736556 |  |
|  |  |  | rs979218 | C | A | -0.04303 | 0.009223 | 3.07E-06 | -0.01298 | 0.019794 | 0.512098 |  |
| Viral infection | Vitamin A | 22.751 | rs10800395 | A | G | -0.08721 | 0.01767 | 7.98E-07 | 0.025338 | 0.017877 | 0.156379 |  |
|  |  |  | rs111423934 | A | G | 0.163191 | 0.035685 | 4.80E-06 | 0.006372 | 0.038325 | 0.867956 |  |
|  |  |  | rs143534274 | A | G | 0.134989 | 0.028799 | 2.77E-06 | 0.049674 | 0.043742 | 0.256113 |  |
|  |  |  | rs146813689 | C | G | -0.37056 | 0.080987 | 4.75E-06 | -0.04592 | 0.073809 | 0.533877 |  |
|  |  |  | rs2028196 | C | T | 0.089132 | 0.019157 | 3.28E-06 | 0.035047 | 0.019883 | 0.077961 |  |
|  |  |  | rs59176398 | C | T | -0.20794 | 0.045146 | 4.10E-06 | 0.003984 | 0.033171 | 0.904408 |  |
|  |  |  | rs72703203 | A | G | -0.28373 | 0.059534 | 1.88E-06 | 0.047475 | 0.038515 | 0.217711 |  |
|  |  |  | rs73035080 | A | G | 0.102195 | 0.019229 | 1.07E-07 | 0.026099 | 0.023255 | 0.261728 |  |
| Viral infection | Vitamin B6 |  | rs10800395 | A | G | -0.08721 | 0.01767 | 7.98E-07 | 0.00317 | 0.006496 | 0.630001 |  |
|  |  |  | rs111423934 | A | G | 0.163191 | 0.035685 | 4.80E-06 | 0.037476 | 0.013222 | 0.0046 |  |
|  |  |  | rs143534274 | A | G | 0.134989 | 0.028799 | 2.77E-06 | 0.01446 | 0.016182 | 0.37 |  |
|  |  |  | rs146813689 | C | G | -0.37056 | 0.080987 | 4.75E-06 | -0.0201 | 0.027834 | 0.47 |  |
|  |  |  | rs2028196 | C | T | 0.089132 | 0.019157 | 3.28E-06 | -0.00597 | 0.007136 | 0.4 |  |
|  |  |  | rs59176398 | C | T | -0.20794 | 0.045146 | 4.10E-06 | 0.010292 | 0.011728 | 0.38 |  |
|  |  |  | rs72703203 | A | G | -0.28373 | 0.059534 | 1.88E-06 | -0.00102 | 0.013632 | 0.94 |  |
|  |  |  | rs73035080 | A | G | 0.102195 | 0.019229 | 1.07E-07 | 0.001044 | 0.00828 | 0.9 |  |
| Viral infection | Vitamin B12 |  | rs10800395 | A | G | -0.08721 | 0.01767 | 7.98E-07 | 0.003778 | 0.006521 | 0.56 |  |
|  |  |  | rs111423934 | A | G | 0.163191 | 0.035685 | 4.80E-06 | -0.00999 | 0.013273 | 0.450001 |  |
|  |  |  | rs143534274 | A | G | 0.134989 | 0.028799 | 2.77E-06 | 0.012905 | 0.016245 | 0.43 |  |
|  |  |  | rs146813689 | C | G | -0.37056 | 0.080987 | 4.75E-06 | 0.007078 | 0.027942 | 0.8 |  |
|  |  |  | rs2028196 | C | T | 0.089132 | 0.019157 | 3.28E-06 | -0.01158 | 0.007165 | 0.11 |  |
|  |  |  | rs59176398 | C | T | -0.20794 | 0.045146 | 4.10E-06 | 0.000988 | 0.011773 | 0.93 |  |
|  |  |  | rs72703203 | A | G | -0.28373 | 0.059534 | 1.88E-06 | 0.004002 | 0.013685 | 0.77 |  |
|  |  |  | rs73035080 | A | G | 0.102195 | 0.019229 | 1.07E-07 | -0.00516 | 0.008311 | 0.53 |  |
| Viral infection | Vitamin C |  | rs10800395 | A | G | -0.08721 | 0.01767 | 7.98E-07 | 0.012422 | 0.006555 | 0.058 |  |
|  |  |  | rs111423934 | A | G | 0.163191 | 0.035685 | 4.80E-06 | 0.009506 | 0.013344 | 0.48 |  |
|  |  |  | rs143534274 | A | G | 0.134989 | 0.028799 | 2.77E-06 | 0.011291 | 0.016332 | 0.49 |  |
|  |  |  | rs146813689 | C | G | -0.37056 | 0.080987 | 4.75E-06 | -0.00715 | 0.028093 | 0.8 |  |
|  |  |  | rs2028196 | C | T | 0.089132 | 0.019157 | 3.28E-06 | -0.00832 | 0.0072 | 0.25 |  |
|  |  |  | rs59176398 | C | T | -0.20794 | 0.045146 | 4.10E-06 | 0.004447 | 0.011836 | 0.709999 |  |
|  |  |  | rs72703203 | A | G | -0.28373 | 0.059534 | 1.88E-06 | -0.02155 | 0.013756 | 0.12 |  |
|  |  |  | rs73035080 | A | G | 0.102195 | 0.019229 | 1.07E-07 | 0.004941 | 0.008356 | 0.55 |  |
| Viral infection | Vitamin D |  | rs10800395 | A | G | -0.08721 | 0.01767 | 7.98E-07 | 0.00800018 | 0.006548 | 0.22 |  |
|  |  |  | rs111423934 | A | G | 0.163191 | 0.035685 | 4.80E-06 | 0.00148793 | 0.013327 | 0.91 |  |
|  |  |  | rs143534274 | A | G | 0.134989 | 0.028799 | 2.77E-06 | 0.00723041 | 0.01631 | 0.66 |  |
|  |  |  | rs146813689 | C | G | -0.37056 | 0.080987 | 4.75E-06 | 0.0141674 | 0.028055 | 0.61 |  |
|  |  |  | rs2028196 | C | T | 0.089132 | 0.019157 | 3.28E-06 | -0.0034364 | 0.007193 | 0.630001 |  |
|  |  |  | rs59176398 | C | T | -0.20794 | 0.045146 | 4.10E-06 | -8.52E-05 | 0.01182 | 0.99 |  |
|  |  |  | rs72703203 | A | G | -0.28373 | 0.059534 | 1.88E-06 | 0.0180558 | 0.013741 | 0.19 |  |
|  |  |  | rs73035080 | A | G | 0.102195 | 0.019229 | 1.07E-07 | 0.00884332 | 0.008345 | 0.29 |  |
| Viral infection | 25(OH)D |  | rs10800395 | A | G | -0.08721 | 0.01767 | 7.98E-07 | -0.0013 | 0.002421 | 0.592107 |  |
|  |  |  | rs143534274 | A | G | 0.134989 | 0.028799 | 2.77E-06 | -0.00101 | 0.006243 | 0.871019 |  |
|  |  |  | rs2028196 | C | T | 0.089132 | 0.019157 | 3.28E-06 | 0.003776 | 0.002676 | 0.158272 |  |
|  |  |  | rs59176398 | C | T | -0.20794 | 0.045146 | 4.10E-06 | -0.00194 | 0.004434 | 0.661428 |  |
|  |  |  | rs73035080 | A | G | 0.102195 | 0.019229 | 1.07E-07 | -0.00556 | 0.003075 | 0.070795 |  |
| Viral infection | Vitamin E |  | rs10800395 | A | G | -0.08721 | 0.01767 | 7.98E-07 | 0.011299 | 0.006546 | 0.084 |  |
|  |  |  | rs111423934 | A | G | 0.163191 | 0.035685 | 4.80E-06 | 0.011904 | 0.013326 | 0.37 |  |
|  |  |  | rs143534274 | A | G | 0.134989 | 0.028799 | 2.77E-06 | 0.025676 | 0.01631 | 0.12 |  |
|  |  |  | rs146813689 | C | G | -0.37056 | 0.080987 | 4.75E-06 | -0.00252 | 0.028055 | 0.93 |  |
|  |  |  | rs2028196 | C | T | 0.089132 | 0.019157 | 3.28E-06 | -0.0114 | 0.007192 | 0.11 |  |
|  |  |  | rs59176398 | C | T | -0.20794 | 0.045146 | 4.10E-06 | 0.009209 | 0.01182 | 0.44 |  |
|  |  |  | rs72703203 | A | G | -0.28373 | 0.059534 | 1.88E-06 | 0.011567 | 0.013737 | 0.4 |  |
|  |  |  | rs73035080 | A | G | 0.102195 | 0.019229 | 1.07E-07 | 0.00857 | 0.008344 | 0.3 |  |
| Vitamin A | Bacterial infection | 25.106 | rs10802946 | T | C | 0.073226 | 0.015446 | 2.13E-06 | 0.010779 | 0.007333 | 0.141595 |  |
|  |  |  | rs11187539 | A | G | -0.07758 | 0.015744 | 8.32E-07 | 0.021511 | 0.007545 | 0.004357 |  |
|  |  |  | rs115971394 | T | C | 0.276481 | 0.057893 | 1.79E-06 | -0.01192 | 0.050381 | 0.812904 |  |
|  |  |  | rs11604481 | C | A | -0.08214 | 0.017827 | 4.07E-06 | -0.00898 | 0.008724 | 0.303529 |  |
|  |  |  | rs11624628 | T | C | -0.27188 | 0.059039 | 4.12E-06 | -0.03816 | 0.042862 | 0.373266 |  |
|  |  |  | rs144434711 | C | G | 0.232473 | 0.048348 | 1.52E-06 | -0.01567 | 0.015655 | 0.316796 |  |
|  |  |  | rs1667226 | T | A | 0.101271 | 0.015461 | 5.74E-11 | 0.000747 | 0.007273 | 0.918147 |  |
|  |  |  | rs1795977 | C | G | -0.12805 | 0.027901 | 4.44E-06 | 0.005002 | 0.009031 | 0.579644 |  |
|  |  |  | rs1883711 | C | G | -0.27778 | 0.045435 | 9.73E-10 | 0.009651 | 0.015127 | 0.52348 |  |
|  |  |  | rs2505415 | C | G | 0.081289 | 0.016912 | 1.54E-06 | 0.016004 | 0.007832 | 0.041006 |  |
|  |  |  | rs3752802 | A | G | 0.081945 | 0.016972 | 1.38E-06 | 0.002293 | 0.009178 | 0.802693 |  |
|  |  |  | rs4665985 | C | A | 0.080474 | 0.017274 | 3.18E-06 | -0.00265 | 0.008583 | 0.75793 |  |
|  |  |  | rs6831628 | G | A | 0.098467 | 0.021092 | 3.03E-06 | 0.010281 | 0.009809 | 0.294581 |  |
| Vitamin B6 | Bacterial infection | 23.218 | rs10138490 | C | T | -0.0535 | 0.011414 | 2.77E-06 | -0.00646 | 0.01515 | 0.669954 |  |
|  |  |  | rs12198456 | T | C | 0.091953 | 0.019723 | 3.13E-06 | -0.02401 | 0.04186 | 0.566205 |  |
|  |  |  | rs12226112 | T | G | 0.028341 | 0.005727 | 7.46E-07 | -0.00394 | 0.007375 | 0.592905 |  |
|  |  |  | rs12412051 | C | G | 0.070864 | 0.014977 | 2.23E-06 | 0.000851 | 0.017731 | 0.961713 |  |
|  |  |  | rs141933624 | A | G | -0.08973 | 0.019281 | 3.26E-06 | 0.019002 | 0.030709 | 0.536066 |  |
|  |  |  | rs155599 | C | T | 0.034253 | 0.005985 | 1.04E-08 | 0.016096 | 0.008068 | 0.046043 |  |
|  |  |  | rs183178622 | T | C | -0.09895 | 0.020684 | 1.72E-06 | 0.040563 | 0.027905 | 0.146055 |  |
|  |  |  | rs188211816 | A | G | -0.07862 | 0.016281 | 1.37E-06 | -0.01692 | 0.018651 | 0.364317 |  |
|  |  |  | rs34938615 | G | A | -0.12202 | 0.026442 | 3.93E-06 | -0.04899 | 0.063255 | 0.438632 |  |
|  |  |  | rs3745438 | C | T | -0.07127 | 0.015597 | 4.89E-06 | 0.003101 | 0.01177 | 0.792157 |  |
|  |  |  | rs3772928 | C | T | -0.02924 | 0.005534 | 1.26E-07 | -0.01056 | 0.007495 | 0.158833 |  |
|  |  |  | rs67450584 | T | C | 0.036705 | 0.007457 | 8.57E-07 | 0.014191 | 0.011154 | 0.203254 |  |
|  |  |  | rs7205927 | C | A | -0.02579 | 0.005535 | 3.18E-06 | 0.005074 | 0.007379 | 0.491688 |  |
|  |  |  | rs7292147 | C | G | -0.02584 | 0.005506 | 2.70E-06 | 0.003926 | 0.00732 | 0.591702 |  |
|  |  |  | rs74640671 | T | C | -0.12718 | 0.02738 | 3.40E-06 | -0.10149 | 0.050281 | 0.043553 |  |
|  |  |  | rs77806858 | C | T | -0.05051 | 0.01061 | 1.93E-06 | -0.00111 | 0.015855 | 0.944363 |  |
|  |  |  | rs9560457 | T | C | 0.025504 | 0.00554 | 4.15E-06 | 0.000611 | 0.007454 | 0.93467 |  |
| Vitamin B12 | Bacterial infection | 22.637 | rs10749205 | C | T | 0.031561 | 0.006837 | 3.91E-06 | 0.006422 | 0.008425 | 0.445929 |  |
|  |  |  | rs10924919 | T | C | -0.02863 | 0.005642 | 3.90E-07 | -0.00456 | 0.007525 | 0.544282 |  |
|  |  |  | rs112961770 | C | G | -0.08885 | 0.018432 | 1.43E-06 | -0.27296 | 0.164791 | 0.097641 |  |
|  |  |  | rs12776611 | A | G | -0.08781 | 0.018896 | 3.36E-06 | -0.02486 | 0.025012 | 0.320167 |  |
|  |  |  | rs148901823 | G | A | -0.04863 | 0.010052 | 1.31E-06 | 0.009021 | 0.012908 | 0.484635 |  |
|  |  |  | rs193228340 | C | T | 0.156303 | 0.034011 | 4.31E-06 | -0.00157 | 0.082869 | 0.984898 |  |
|  |  |  | rs388561 | C | T | 0.040271 | 0.008549 | 2.47E-06 | 0.001939 | 0.010705 | 0.856285 |  |
|  |  |  | rs61994378 | C | T | 0.093456 | 0.019774 | 2.29E-06 | -0.00159 | 0.022336 | 0.94323 |  |
|  |  |  | rs67568068 | C | T | -0.03174 | 0.006647 | 1.80E-06 | -0.0054 | 0.007984 | 0.498847 |  |
| Vitamin C | Bacterial infection | 23.208 | rs114598078 | T | C | 0.065578 | 0.013764 | 1.89E-06 | -0.00892 | 0.01985 | 0.653326 |  |
|  |  |  | rs11650824 | A | T | 0.079479 | 0.015882 | 5.61E-07 | 0.004015 | 0.013953 | 0.773567 |  |
|  |  |  | rs17482258 | T | C | 0.042829 | 0.00926 | 3.75E-06 | -0.0129 | 0.011451 | 0.260031 |  |
|  |  |  | rs1883993 | A | G | 0.044959 | 0.009353 | 1.53E-06 | -0.0204 | 0.008869 | 0.021428 |  |
|  |  |  | rs4238567 | C | T | 0.025307 | 0.005508 | 4.34E-06 | 0.002371 | 0.007322 | 0.74611 |  |
|  |  |  | rs4481190 | C | A | -0.03064 | 0.005744 | 9.61E-08 | 0.019576 | 0.007546 | 0.009478 |  |
|  |  |  | rs61868302 | T | C | -0.0571 | 0.011839 | 1.41E-06 | -0.03625 | 0.019161 | 0.058487 |  |
|  |  |  | rs74978963 | T | C | 0.150814 | 0.031021 | 1.16E-06 | -0.02604 | 0.02267 | 0.25068 |  |
|  |  |  | rs7626478 | A | G | 0.02798 | 0.006101 | 4.52E-06 | 0.007883 | 0.008392 | 0.347601 |  |
|  |  |  | rs9540734 | A | G | -0.02593 | 0.005485 | 2.27E-06 | 0.011595 | 0.00728 | 0.111209 |  |
| Vitamin D | Bacterial infection | 22.762 | rs10469075 | T | C | -0.03246 | 0.007013 | 3.70E-06 | 0.005942 | 0.009954 | 0.550554 |  |
|  |  |  | rs10469076 | A | C | -0.03156 | 0.006908 | 4.90E-06 | 0.006965 | 0.009772 | 0.475973 |  |
|  |  |  | rs11692645 | C | G | -0.05899 | 0.012798 | 4.00E-06 | -0.00535 | 0.01622 | 0.741517 |  |
|  |  |  | rs117693112 | A | G | 0.068243 | 0.014479 | 2.40E-06 | 0.010347 | 0.027046 | 0.702047 |  |
|  |  |  | rs148901823 | G | A | -0.04614 | 0.010092 | 4.80E-06 | 0.009021 | 0.012908 | 0.484635 |  |
|  |  |  | rs17301981 | C | T | -0.04379 | 0.009585 | 4.90E-06 | 0.007461 | 0.0129 | 0.563023 |  |
|  |  |  | rs186610469 | C | T | 0.180487 | 0.033808 | 9.40E-08 | -0.05412 | 0.039257 | 0.168007 |  |
|  |  |  | rs191065076 | G | A | 0.210081 | 0.038238 | 3.90E-08 | 0.034311 | 0.040529 | 0.397238 |  |
|  |  |  | rs192447369 | A | G | 0.216917 | 0.038487 | 1.70E-08 | 0.034403 | 0.040529 | 0.395962 |  |
|  |  |  | rs2459139 | C | A | -0.0342 | 0.007084 | 1.40E-06 | -0.01433 | 0.009877 | 0.146755 |  |
|  |  |  | rs2673883 | C | T | -0.03429 | 0.007054 | 1.20E-06 | -0.01323 | 0.009849 | 0.17904 |  |
|  |  |  | rs2687869 | A | G | -0.03363 | 0.007054 | 1.90E-06 | -0.01329 | 0.00986 | 0.177683 |  |
|  |  |  | rs35775421 | A | G | -0.05597 | 0.012219 | 4.60E-06 | -0.01641 | 0.012364 | 0.184462 |  |
|  |  |  | rs57038272 | T | C | 0.033332 | 0.007237 | 4.10E-06 | 0.002029 | 0.008908 | 0.819814 |  |
|  |  |  | rs582962 | A | G | -0.02832 | 0.006081 | 3.20E-06 | -0.00809 | 0.007461 | 0.277933 |  |
|  |  |  | rs61942184 | C | G | 0.082217 | 0.017757 | 3.70E-06 | -0.0094 | 0.026443 | 0.722134 |  |
|  |  |  | rs62329691 | T | C | 0.120492 | 0.025884 | 3.20E-06 | -0.03199 | 0.066075 | 0.62823 |  |
|  |  |  | rs679830 | C | T | -0.06018 | 0.012755 | 2.40E-06 | 0.010376 | 0.01566 | 0.507588 |  |
|  |  |  | rs7076199 | A | G | 0.036138 | 0.007896 | 4.70E-06 | -0.00942 | 0.011882 | 0.428039 |  |
|  |  |  | rs7096312 | G | A | 0.036244 | 0.007901 | 4.50E-06 | -0.00913 | 0.011895 | 0.442558 |  |
|  |  |  | rs74593039 | C | G | 0.035005 | 0.007539 | 3.40E-06 | -0.0094 | 0.01021 | 0.357274 |  |
|  |  |  | rs75145722 | C | G | -0.04415 | 0.009194 | 1.60E-06 | -0.00046 | 0.009363 | 0.960707 |  |
|  |  |  | rs75713989 | T | C | 0.039147 | 0.008167 | 1.60E-06 | 0.024783 | 0.011395 | 0.029634 |  |
|  |  |  | rs77503650 | T | C | 0.150799 | 0.032064 | 2.60E-06 | 0.03284 | 0.040084 | 0.412626 |  |
|  |  |  | rs78242607 | T | G | 0.036548 | 0.007911 | 3.80E-06 | -0.00933 | 0.011896 | 0.432669 |  |
|  |  |  | rs80261862 | T | C | -0.04487 | 0.009267 | 1.30E-06 | 0.001393 | 0.009425 | 0.882521 |  |
|  |  |  | rs9328367 | T | A | 0.02618 | 0.005617 | 3.10E-06 | 0.00472 | 0.007313 | 0.518707 |  |
| 25(OH)D | Bacterial infection | 81.88 | rs10070734 | T | C | -0.01083 | 0.002237 | 1.29E-06 | 0.007513 | 0.008194 | 0.359245 |  |
|  |  |  | rs10090648 | A | G | -0.01488 | 0.00312 | 1.86E-06 | -0.01785 | 0.012547 | 0.15487 |  |
|  |  |  | rs10121187 | G | C | 0.00931 | 0.002036 | 4.81E-06 | -0.00549 | 0.007282 | 0.450679 |  |
|  |  |  | rs10171594 | G | A | 0.01376 | 0.002705 | 3.62E-07 | -0.00469 | 0.008185 | 0.566986 |  |
|  |  |  | rs10277163 | G | A | -0.01433 | 0.002351 | 1.08E-09 | -0.00883 | 0.008682 | 0.308966 |  |
|  |  |  | rs1038165 | C | T | -0.01151 | 0.002056 | 2.15E-08 | -0.01423 | 0.007345 | 0.052649 |  |
|  |  |  | rs1042034 | C | T | 0.015125 | 0.0025 | 1.45E-09 | -0.0099 | 0.00819 | 0.226577 |  |
|  |  |  | rs10438978 | T | C | 0.017224 | 0.002644 | 7.34E-11 | 0.00217 | 0.009472 | 0.81877 |  |
|  |  |  | rs1047891 | A | C | -0.0134 | 0.00218 | 7.96E-10 | -0.00779 | 0.007796 | 0.317698 |  |
|  |  |  | rs1048328 | A | G | 0.03135 | 0.003744 | 5.58E-17 | 0.001951 | 0.012173 | 0.872659 |  |
|  |  |  | rs10521222 | T | C | 0.025151 | 0.004857 | 2.24E-07 | -0.00472 | 0.023114 | 0.83835 |  |
|  |  |  | rs10822184 | C | T | -0.00992 | 0.002029 | 1.01E-06 | 0.000952 | 0.007287 | 0.896018 |  |
|  |  |  | rs10859995 | T | C | 0.043626 | 0.002055 | 4.60E-100 | -0.00267 | 0.00767 | 0.728217 |  |
|  |  |  | rs10896045 | A | G | -0.01278 | 0.002229 | 9.81E-09 | 0.0068 | 0.008144 | 0.403729 |  |
|  |  |  | rs10952882 | A | G | 0.0098 | 0.002042 | 1.58E-06 | 0.014927 | 0.007309 | 0.041134 |  |
|  |  |  | rs11060406 | T | C | -0.03022 | 0.005581 | 6.10E-08 | -0.00827 | 0.016828 | 0.62293 |  |
|  |  |  | rs112072036 | G | A | -0.20594 | 0.004981 | 0 | -0.00579 | 0.019282 | 0.763845 |  |
|  |  |  | rs11264361 | G | T | 0.017488 | 0.002341 | 7.97E-14 | 0.005808 | 0.008134 | 0.475229 |  |
|  |  |  | rs1128535 | C | T | -0.01641 | 0.002029 | 6.06E-16 | -0.00461 | 0.007561 | 0.54205 |  |
|  |  |  | rs115288876 | A | G | 0.078806 | 0.004982 | 2.36E-56 | -0.00039 | 0.018803 | 0.983315 |  |
|  |  |  | rs11542462 | A | G | -0.02478 | 0.002983 | 9.72E-17 | 0.006396 | 0.012501 | 0.608924 |  |
|  |  |  | rs11625899 | T | C | 0.009892 | 0.002124 | 3.21E-06 | -0.00681 | 0.007525 | 0.365407 |  |
|  |  |  | rs11637664 | T | A | -0.01033 | 0.002036 | 3.92E-07 | 0.001296 | 0.007278 | 0.858669 |  |
|  |  |  | rs11651767 | A | G | 0.009827 | 0.002141 | 4.42E-06 | -0.00412 | 0.007506 | 0.582856 |  |
|  |  |  | rs11745926 | C | T | -0.01073 | 0.002339 | 4.48E-06 | 0.005338 | 0.007892 | 0.498786 |  |
|  |  |  | rs11752007 | C | T | 0.016456 | 0.003074 | 8.61E-08 | 0.018652 | 0.011681 | 0.110318 |  |
|  |  |  | rs117862422 | C | T | -0.05046 | 0.008655 | 5.52E-09 | 0.021249 | 0.018263 | 0.24464 |  |
|  |  |  | rs11791258 | A | G | 0.014081 | 0.002581 | 4.85E-08 | -0.01779 | 0.010817 | 0.100123 |  |
|  |  |  | rs11865670 | G | A | 0.010082 | 0.002138 | 2.42E-06 | -0.00397 | 0.007279 | 0.585651 |  |
|  |  |  | rs11867297 | T | C | 0.013543 | 0.002095 | 1.01E-10 | 0.001609 | 0.007664 | 0.833705 |  |
|  |  |  | rs11898833 | C | T | 0.011351 | 0.002344 | 1.28E-06 | -0.00815 | 0.008576 | 0.341886 |  |
|  |  |  | rs12056768 | T | G | 0.023196 | 0.002064 | 2.65E-29 | -0.00304 | 0.00727 | 0.676153 |  |
|  |  |  | rs12147536 | C | T | -0.01354 | 0.002822 | 1.61E-06 | -0.00741 | 0.010065 | 0.461897 |  |
|  |  |  | rs12369406 | T | C | 0.009818 | 0.002098 | 2.88E-06 | -0.00595 | 0.007625 | 0.435159 |  |
|  |  |  | rs12403824 | G | C | 0.010675 | 0.002221 | 1.54E-06 | 0.003649 | 0.007685 | 0.634976 |  |
|  |  |  | rs12422983 | T | C | 0.014123 | 0.00291 | 1.22E-06 | -0.00325 | 0.010732 | 0.761753 |  |
|  |  |  | rs12462826 | A | G | -0.01321 | 0.002115 | 4.18E-10 | -0.00321 | 0.007712 | 0.677553 |  |
|  |  |  | rs1260326 | T | C | -0.01972 | 0.002074 | 1.96E-21 | 0.004641 | 0.007627 | 0.542896 |  |
|  |  |  | rs12616170 | C | A | -0.00999 | 0.002174 | 4.32E-06 | 0.002227 | 0.007652 | 0.771045 |  |
|  |  |  | rs12713125 | G | T | -0.01122 | 0.002401 | 2.94E-06 | -0.01231 | 0.009021 | 0.172327 |  |
|  |  |  | rs12775091 | T | C | 0.015562 | 0.002477 | 3.33E-10 | -0.00386 | 0.008001 | 0.629814 |  |
|  |  |  | rs12816349 | A | G | 0.011015 | 0.002372 | 3.42E-06 | 0.010851 | 0.009386 | 0.247658 |  |
|  |  |  | rs12919213 | T | C | -0.01495 | 0.002875 | 1.99E-07 | -0.00529 | 0.011282 | 0.638937 |  |
|  |  |  | rs13064022 | A | G | 0.019893 | 0.004222 | 2.46E-06 | -0.00482 | 0.012726 | 0.704812 |  |
|  |  |  | rs13076508 | C | T | 0.025054 | 0.00451 | 2.78E-08 | 0.005913 | 0.01819 | 0.745135 |  |
|  |  |  | rs13201062 | A | C | 0.010002 | 0.002073 | 1.40E-06 | -0.00297 | 0.007294 | 0.684253 |  |
|  |  |  | rs1321247 | T | A | -0.02218 | 0.003366 | 4.36E-11 | 0.024979 | 0.011495 | 0.029772 |  |
|  |  |  | rs1324190 | T | C | 0.010232 | 0.002099 | 1.09E-06 | -0.00408 | 0.007363 | 0.579385 |  |
|  |  |  | rs13278404 | C | G | -0.01416 | 0.002942 | 1.48E-06 | -0.01567 | 0.013334 | 0.239889 |  |
|  |  |  | rs13294734 | T | C | 0.012568 | 0.002058 | 1.02E-09 | 0.008961 | 0.007273 | 0.21793 |  |
|  |  |  | rs13389628 | T | C | 0.02011 | 0.003934 | 3.20E-07 | 0.010672 | 0.013951 | 0.44431 |  |
|  |  |  | rs1343776 | A | G | 0.018076 | 0.00245 | 1.62E-13 | 0.002625 | 0.008784 | 0.765049 |  |
|  |  |  | rs1369136 | A | G | -0.02216 | 0.004615 | 1.58E-06 | 0.013822 | 0.015201 | 0.363201 |  |
|  |  |  | rs138335 | C | G | 0.013766 | 0.002151 | 1.56E-10 | 0.007359 | 0.007505 | 0.326806 |  |
|  |  |  | rs1384687 | A | G | -0.01687 | 0.002997 | 1.82E-08 | 0.007743 | 0.008271 | 0.349231 |  |
|  |  |  | rs1395975 | A | G | 0.00994 | 0.002053 | 1.28E-06 | 0.002567 | 0.007331 | 0.72627 |  |
|  |  |  | rs141335934 | T | C | 0.01837 | 0.003943 | 3.18E-06 | 0.013234 | 0.012218 | 0.278726 |  |
|  |  |  | rs142004400 | C | A | -0.031 | 0.005596 | 3.01E-08 | 0.031657 | 0.025953 | 0.22255 |  |
|  |  |  | rs142158911 | A | G | 0.026284 | 0.003234 | 4.43E-16 | -0.01709 | 0.012029 | 0.155515 |  |
|  |  |  | rs1432902 | A | C | -0.02036 | 0.004251 | 1.67E-06 | 0.025575 | 0.0149 | 0.08608 |  |
|  |  |  | rs1460665 | G | A | -0.01486 | 0.003173 | 2.82E-06 | -0.00285 | 0.010666 | 0.789015 |  |
|  |  |  | rs1532085 | A | G | -0.02528 | 0.002086 | 8.60E-34 | 0.002065 | 0.007354 | 0.778927 |  |
|  |  |  | rs1548904 | A | C | -0.00997 | 0.002076 | 1.56E-06 | 0.009288 | 0.007365 | 0.207273 |  |
|  |  |  | rs1558902 | A | T | 0.011521 | 0.002066 | 2.48E-08 | 0.025316 | 0.007334 | 0.000557 |  |
|  |  |  | rs1664781 | G | A | 0.010737 | 0.0022 | 1.06E-06 | 0.002304 | 0.00771 | 0.765064 |  |
|  |  |  | rs1684600 | T | C | -0.01253 | 0.002217 | 1.59E-08 | ####### | 0.008361 | 0.992982 |  |
|  |  |  | rs16848096 | G | T | 0.017428 | 0.003571 | 1.06E-06 | 0.012582 | 0.011207 | 0.261599 |  |
|  |  |  | rs16879122 | G | A | 0.017407 | 0.003812 | 4.96E-06 | 0.013769 | 0.011822 | 0.244151 |  |
|  |  |  | rs17057661 | C | T | 0.010927 | 0.002146 | 3.57E-07 | -0.00102 | 0.007825 | 0.895891 |  |
|  |  |  | rs17105586 | G | T | 0.009942 | 0.002112 | 2.52E-06 | -0.01241 | 0.00745 | 0.09566 |  |
|  |  |  | rs1714336 | T | C | 0.014427 | 0.002082 | 4.23E-12 | 0.01236 | 0.007466 | 0.097839 |  |
|  |  |  | rs17207784 | C | T | -0.01349 | 0.002171 | 5.14E-10 | -0.01109 | 0.007824 | 0.156318 |  |
|  |  |  | rs17216177 | C | T | 0.02044 | 0.004368 | 2.88E-06 | -0.02363 | 0.023016 | 0.304585 |  |
|  |  |  | rs17593743 | C | A | 0.015751 | 0.003252 | 1.28E-06 | -0.01388 | 0.014243 | 0.329781 |  |
|  |  |  | rs17688247 | T | C | -0.01325 | 0.002872 | 3.95E-06 | -0.00147 | 0.008846 | 0.867581 |  |
|  |  |  | rs1792287 | G | A | 0.023152 | 0.002307 | 1.06E-23 | -0.0065 | 0.007998 | 0.416022 |  |
|  |  |  | rs1800588 | T | C | -0.0305 | 0.002469 | 4.73E-35 | 0.004742 | 0.008411 | 0.572925 |  |
|  |  |  | rs1800775 | A | C | -0.01742 | 0.002029 | 9.23E-18 | 0.000953 | 0.007248 | 0.895372 |  |
|  |  |  | rs1858889 | A | C | -0.01345 | 0.002031 | 3.49E-11 | 0.003438 | 0.007274 | 0.636483 |  |
|  |  |  | rs1871395 | G | A | -0.02037 | 0.002827 | 5.72E-13 | -0.00241 | 0.008128 | 0.766913 |  |
|  |  |  | rs1894100 | T | G | -0.10215 | 0.002592 | 0 | 0.010079 | 0.007603 | 0.184967 |  |
|  |  |  | rs1949633 | T | C | -0.01142 | 0.002086 | 4.45E-08 | -0.00455 | 0.007581 | 0.548825 |  |
|  |  |  | rs1993116 | A | G | 0.084686 | 0.00206 | 0 | -0.0046 | 0.007319 | 0.529794 |  |
|  |  |  | rs2037511 | A | G | 0.017662 | 0.002727 | 9.41E-11 | -0.01355 | 0.009873 | 0.169828 |  |
|  |  |  | rs2069502 | T | C | -0.01166 | 0.002172 | 7.93E-08 | -0.01532 | 0.007657 | 0.045395 |  |
|  |  |  | rs2070737 | T | A | -0.01062 | 0.002112 | 4.89E-07 | 0.000608 | 0.007955 | 0.93907 |  |
|  |  |  | rs2074735 | C | G | 0.029267 | 0.00412 | 1.22E-12 | -0.00358 | 0.010472 | 0.732767 |  |
|  |  |  | rs2157829 | A | G | 0.009993 | 0.002166 | 3.94E-06 | 0.013767 | 0.008368 | 0.099933 |  |
|  |  |  | rs2165331 | G | T | -0.01009 | 0.002207 | 4.79E-06 | -0.00275 | 0.007997 | 0.731242 |  |
|  |  |  | rs2229742 | C | G | -0.02498 | 0.003314 | 4.75E-14 | 0.013018 | 0.011569 | 0.260502 |  |
|  |  |  | rs2239537 | T | G | -0.01242 | 0.002368 | 1.55E-07 | 0.002634 | 0.008791 | 0.764483 |  |
|  |  |  | rs2297991 | T | C | -0.01275 | 0.002256 | 1.57E-08 | 0.003649 | 0.007681 | 0.634686 |  |
|  |  |  | rs2336865 | C | T | 0.010225 | 0.002111 | 1.28E-06 | -0.00585 | 0.00768 | 0.446095 |  |
|  |  |  | rs2470937 | A | T | -0.01105 | 0.002042 | 6.30E-08 | 0.008386 | 0.007342 | 0.253342 |  |
|  |  |  | rs2494429 | A | G | 0.014846 | 0.002673 | 2.80E-08 | -0.00033 | 0.00958 | 0.972858 |  |
|  |  |  | rs2595644 | T | G | -0.01226 | 0.002097 | 4.97E-09 | 0.011331 | 0.007661 | 0.139126 |  |
|  |  |  | rs2756119 | A | G | 0.012143 | 0.00211 | 8.71E-09 | 0.008063 | 0.007487 | 0.281555 |  |
|  |  |  | rs2784773 | T | C | 0.010925 | 0.002129 | 2.87E-07 | 0.014657 | 0.0079 | 0.063548 |  |
|  |  |  | rs2807834 | T | G | 0.015063 | 0.002187 | 5.66E-12 | 0.006489 | 0.008105 | 0.423331 |  |
|  |  |  | rs28435470 | G | A | 0.01187 | 0.002148 | 3.29E-08 | -0.00971 | 0.007379 | 0.188336 |  |
|  |  |  | rs2847500 | A | G | -0.02255 | 0.003087 | 2.77E-13 | -0.00165 | 0.010315 | 0.872834 |  |
|  |  |  | rs296381 | C | T | 0.061106 | 0.002763 | 2.30E-108 | -0.00568 | 0.010169 | 0.576731 |  |
|  |  |  | rs2977273 | G | A | -0.0115 | 0.002241 | 2.84E-07 | -0.00634 | 0.00794 | 0.424817 |  |
|  |  |  | rs317195 | C | T | -0.01437 | 0.003103 | 3.66E-06 | -0.00243 | 0.008475 | 0.774487 |  |
|  |  |  | rs325393 | T | G | -0.01365 | 0.002277 | 2.03E-09 | 0.005487 | 0.007835 | 0.483702 |  |
|  |  |  | rs34177108 | A | C | -0.0115 | 0.002321 | 7.28E-07 | 0.012885 | 0.009095 | 0.156595 |  |
|  |  |  | rs34726834 | T | C | 0.014013 | 0.002349 | 2.42E-09 | -0.00528 | 0.00795 | 0.506322 |  |
|  |  |  | rs35270497 | T | C | 0.015672 | 0.002681 | 5.08E-09 | 0.002581 | 0.008922 | 0.772336 |  |
|  |  |  | rs35285316 | T | C | 0.014437 | 0.002706 | 9.56E-08 | 0.008004 | 0.011025 | 0.467811 |  |
|  |  |  | rs35634730 | T | C | 0.014988 | 0.002993 | 5.52E-07 | -0.00643 | 0.013767 | 0.640622 |  |
|  |  |  | rs35823191 | C | T | -0.02326 | 0.002141 | 1.65E-27 | 0.000461 | 0.007559 | 0.951341 |  |
|  |  |  | rs35947684 | C | G | 0.010118 | 0.00207 | 1.02E-06 | ####### | 0.007286 | 0.998688 |  |
|  |  |  | rs3732220 | A | G | -0.04784 | 0.003633 | 1.31E-39 | -0.00115 | 0.013772 | 0.933394 |  |
|  |  |  | rs3750569 | G | A | 0.013667 | 0.002657 | 2.68E-07 | 0.007317 | 0.009994 | 0.464111 |  |
|  |  |  | rs3768790 | A | G | -0.02032 | 0.003867 | 1.49E-07 | -0.00171 | 0.012262 | 0.889407 |  |
|  |  |  | rs3784441 | A | G | -0.01297 | 0.00244 | 1.06E-07 | -0.00029 | 0.00814 | 0.971445 |  |
|  |  |  | rs3890624 | G | A | 0.010535 | 0.002108 | 5.81E-07 | 0.00318 | 0.007657 | 0.677896 |  |
|  |  |  | rs3924150 | C | T | -0.01126 | 0.002063 | 4.82E-08 | -0.00446 | 0.007323 | 0.542344 |  |
|  |  |  | rs4147536 | A | C | 0.014804 | 0.00249 | 2.76E-09 | -0.00698 | 0.008384 | 0.405406 |  |
|  |  |  | rs4364259 | A | G | 0.017241 | 0.002567 | 1.86E-11 | -0.00804 | 0.0088 | 0.360714 |  |
|  |  |  | rs4441458 | T | C | -0.01069 | 0.002262 | 2.28E-06 | 0.008424 | 0.007839 | 0.282505 |  |
|  |  |  | rs4504191 | T | G | -0.01088 | 0.002341 | 3.33E-06 | -0.00202 | 0.00867 | 0.815456 |  |
|  |  |  | rs4580037 | C | A | -0.01356 | 0.002251 | 1.68E-09 | -0.00218 | 0.00819 | 0.789662 |  |
|  |  |  | rs4678803 | A | G | 0.012135 | 0.00226 | 7.87E-08 | 0.008246 | 0.008484 | 0.331066 |  |
|  |  |  | rs4694423 | A | C | -0.0937 | 0.002056 | 0 | -0.00536 | 0.00734 | 0.465187 |  |
|  |  |  | rs4715218 | G | A | 0.010448 | 0.002222 | 2.57E-06 | 0.014836 | 0.007912 | 0.060781 |  |
|  |  |  | rs4846913 | C | A | -0.01318 | 0.002077 | 2.19E-10 | 0.004259 | 0.007296 | 0.559425 |  |
|  |  |  | rs4849777 | C | A | -0.01253 | 0.00252 | 6.69E-07 | -0.00544 | 0.008882 | 0.540566 |  |
|  |  |  | rs4916928 | C | A | 0.013428 | 0.0029 | 3.65E-06 | 0.001909 | 0.009999 | 0.848568 |  |
|  |  |  | rs4924 | C | T | -0.0104 | 0.002036 | 3.21E-07 | 0.005019 | 0.007372 | 0.49592 |  |
|  |  |  | rs4983329 | C | A | -0.00941 | 0.002046 | 4.22E-06 | -0.00801 | 0.007298 | 0.272712 |  |
|  |  |  | rs5011717 | G | A | 0.009609 | 0.002044 | 2.59E-06 | 0.003176 | 0.007316 | 0.664179 |  |
|  |  |  | rs512083 | C | T | 0.012217 | 0.002043 | 2.23E-09 | 0.007853 | 0.007261 | 0.279478 |  |
|  |  |  | rs55853471 | G | C | -0.05022 | 0.005089 | 5.72E-23 | 0.005508 | 0.019004 | 0.77195 |  |
|  |  |  | rs57601828 | T | A | 0.011542 | 0.002082 | 2.96E-08 | -0.00432 | 0.007288 | 0.553261 |  |
|  |  |  | rs5770794 | T | C | -0.01331 | 0.002211 | 1.74E-09 | 0.001252 | 0.007536 | 0.868024 |  |
|  |  |  | rs59488684 | C | A | 0.01003 | 0.002057 | 1.09E-06 | 0.004084 | 0.007274 | 0.574474 |  |
|  |  |  | rs6001026 | T | C | 0.01039 | 0.002148 | 1.32E-06 | 0.012994 | 0.007667 | 0.090112 |  |
|  |  |  | rs6003465 | C | T | -0.011 | 0.002164 | 3.74E-07 | -0.00782 | 0.008155 | 0.337331 |  |
|  |  |  | rs6129648 | G | A | 0.014063 | 0.002106 | 2.44E-11 | -0.00759 | 0.007708 | 0.324846 |  |
|  |  |  | rs61698755 | T | C | 0.011465 | 0.002051 | 2.25E-08 | 0.009721 | 0.00732 | 0.184207 |  |
|  |  |  | rs61747728 | T | C | 0.030306 | 0.005269 | 8.83E-09 | -0.00403 | 0.015147 | 0.790049 |  |
|  |  |  | rs61813875 | G | C | 0.082129 | 0.006589 | 1.16E-35 | 0.038203 | 0.040703 | 0.347953 |  |
|  |  |  | rs62007299 | G | A | 0.012421 | 0.002244 | 3.12E-08 | 0.013703 | 0.007765 | 0.077616 |  |
|  |  |  | rs62166770 | G | T | 0.011049 | 0.002265 | 1.07E-06 | -0.0039 | 0.009139 | 0.669686 |  |
|  |  |  | rs62319568 | G | A | 0.014434 | 0.002851 | 4.13E-07 | 0.00343 | 0.009466 | 0.717122 |  |
|  |  |  | rs62422641 | A | G | 0.010835 | 0.002329 | 3.30E-06 | -0.00591 | 0.008426 | 0.482902 |  |
|  |  |  | rs6438900 | G | C | 0.015049 | 0.00234 | 1.27E-10 | -0.00079 | 0.00791 | 0.920152 |  |
|  |  |  | rs6599618 | T | C | -0.01005 | 0.002086 | 1.46E-06 | -0.00732 | 0.007308 | 0.316505 |  |
|  |  |  | rs6600876 | G | T | -0.03124 | 0.002036 | 3.94E-53 | 0.010203 | 0.00732 | 0.163391 |  |
|  |  |  | rs6706116 | T | C | 0.010184 | 0.002077 | 9.48E-07 | 0.003834 | 0.007456 | 0.607058 |  |
|  |  |  | rs6775277 | A | G | 0.010629 | 0.002194 | 1.27E-06 | 0.004204 | 0.007583 | 0.579347 |  |
|  |  |  | rs6834488 | T | C | -0.01445 | 0.002059 | 2.26E-12 | 0.003581 | 0.007735 | 0.643403 |  |
|  |  |  | rs6871090 | A | C | 0.010072 | 0.002148 | 2.74E-06 | -0.01078 | 0.007319 | 0.14095 |  |
|  |  |  | rs6877237 | G | C | -0.01161 | 0.002378 | 1.04E-06 | -0.00663 | 0.008113 | 0.413541 |  |
|  |  |  | rs6963 | A | T | -0.01206 | 0.002253 | 8.66E-08 | 0.002949 | 0.008462 | 0.727425 |  |
|  |  |  | rs6972278 | T | G | 0.009663 | 0.002104 | 4.36E-06 | 0.003939 | 0.007456 | 0.597273 |  |
|  |  |  | rs6982486 | G | A | -0.01001 | 0.002151 | 3.28E-06 | -0.00309 | 0.007463 | 0.678347 |  |
|  |  |  | rs703491 | T | C | 0.012157 | 0.002573 | 2.31E-06 | -0.003 | 0.009571 | 0.754095 |  |
|  |  |  | rs7077708 | G | A | -0.01084 | 0.002211 | 9.54E-07 | -0.00798 | 0.007288 | 0.273529 |  |
|  |  |  | rs71601787 | A | G | 0.044836 | 0.002178 | 3.97E-94 | -0.00472 | 0.007961 | 0.553486 |  |
|  |  |  | rs7235662 | A | G | 0.013349 | 0.002911 | 4.53E-06 | -0.01077 | 0.010355 | 0.298492 |  |
|  |  |  | rs727857 | G | A | 0.012055 | 0.002099 | 9.27E-09 | 0.009773 | 0.007301 | 0.180669 |  |
|  |  |  | rs72997688 | G | A | 0.025696 | 0.004374 | 4.24E-09 | 0.007257 | 0.014231 | 0.61008 |  |
|  |  |  | rs73413596 | C | T | 0.022347 | 0.003889 | 9.15E-09 | 0.005333 | 0.015491 | 0.730629 |  |
|  |  |  | rs740772 | A | G | 0.009806 | 0.002102 | 3.08E-06 | 0.013754 | 0.007381 | 0.062391 |  |
|  |  |  | rs742493 | C | T | 0.018353 | 0.003206 | 1.04E-08 | 0.012061 | 0.012299 | 0.326786 |  |
|  |  |  | rs745570 | A | G | 0.010469 | 0.002031 | 2.53E-07 | -0.00145 | 0.007257 | 0.841997 |  |
|  |  |  | rs7569755 | A | G | 0.013639 | 0.002256 | 1.49E-09 | 0.001779 | 0.008839 | 0.840468 |  |
|  |  |  | rs75936148 | A | G | -0.01765 | 0.003532 | 5.76E-07 | -0.01935 | 0.014018 | 0.167561 |  |
|  |  |  | rs7652808 | T | G | 0.021286 | 0.002126 | 1.36E-23 | 0.011945 | 0.008167 | 0.143588 |  |
|  |  |  | rs7657132 | G | A | -0.0136 | 0.002206 | 6.99E-10 | -0.00465 | 0.007836 | 0.552846 |  |
|  |  |  | rs7712001 | G | T | 0.011939 | 0.002062 | 7.05E-09 | -0.0094 | 0.007477 | 0.208687 |  |
|  |  |  | rs77521820 | T | G | 0.020809 | 0.003989 | 1.82E-07 | 0.00754 | 0.013818 | 0.585289 |  |
|  |  |  | rs77532868 | T | C | 0.025956 | 0.004563 | 1.28E-08 | 0.004952 | 0.020614 | 0.810149 |  |
|  |  |  | rs77559960 | T | G | -0.0131 | 0.002535 | 2.39E-07 | -0.01392 | 0.009253 | 0.13257 |  |
|  |  |  | rs7780345 | C | T | -0.01723 | 0.003609 | 1.81E-06 | -0.01564 | 0.013616 | 0.250593 |  |
|  |  |  | rs77924615 | A | G | -0.01525 | 0.00259 | 3.94E-09 | 0.010922 | 0.008753 | 0.212092 |  |
|  |  |  | rs78649910 | A | T | -0.01914 | 0.003322 | 8.33E-09 | 0.004337 | 0.011125 | 0.69663 |  |
|  |  |  | rs7955128 | A | T | -0.01306 | 0.002039 | 1.48E-10 | 0.004678 | 0.007324 | 0.522985 |  |
|  |  |  | rs79687284 | C | G | -0.02721 | 0.005543 | 9.19E-07 | 0.024938 | 0.02766 | 0.367286 |  |
|  |  |  | rs79699939 | C | A | 0.0248 | 0.004392 | 1.64E-08 | 0.021905 | 0.014396 | 0.128094 |  |
|  | |  |  | rs7997035 | T | C | -0.01037 | 0.002102 | 8.11E-07 | -0.00151 | 0.007601 | 0.842859 |
|  |  |  |  | rs8018720 | G | C | 0.034496 | 0.002661 | 1.93E-38 | -0.00326 | 0.010175 | 0.748864 |
|  |  |  |  | rs8026614 | T | C | -0.0097 | 0.002044 | 2.08E-06 | -0.00168 | 0.007265 | 0.817516 |
|  |  |  |  | rs804281 | A | G | -0.0159 | 0.00206 | 1.20E-14 | -0.00292 | 0.00787 | 0.711043 |
|  |  |  |  | rs8063565 | G | C | -0.0115 | 0.002296 | 5.42E-07 | 0.017974 | 0.00799 | 0.024482 |
|  |  |  |  | rs8107974 | T | A | 0.035567 | 0.003823 | 1.36E-20 | -0.00447 | 0.014754 | 0.762079 |
|  |  |  |  | rs8121940 | G | C | -0.04356 | 0.002549 | 1.77E-65 | -0.01079 | 0.008571 | 0.20804 |
|  |  |  |  | rs818168 | C | T | 0.010466 | 0.002151 | 1.14E-06 | 0.002578 | 0.007323 | 0.724866 |
|  |  |  |  | rs8614 | A | C | -0.0143 | 0.002629 | 5.32E-08 | 0.004845 | 0.010084 | 0.630909 |
|  |  |  |  | rs9363415 | T | C | 0.010915 | 0.002033 | 7.93E-08 | 0.000846 | 0.007297 | 0.907708 |
|  |  |  |  | rs9375037 | C | A | 0.011706 | 0.002054 | 1.21E-08 | 0.003052 | 0.007283 | 0.675182 |
|  |  |  |  | rs9409266 | G | A | 0.016777 | 0.002947 | 1.24E-08 | -0.01675 | 0.009657 | 0.082754 |
|  |  |  |  | rs9559508 | T | C | -0.01348 | 0.002616 | 2.54E-07 | -0.00309 | 0.009011 | 0.731863 |
|  |  |  |  | rs964184 | G | C | -0.04068 | 0.002989 | 3.50E-42 | 0.011369 | 0.010286 | 0.269027 |
|  |  |  |  | rs986649 | G | A | 0.012864 | 0.002178 | 3.51E-09 | 0.014763 | 0.00748 | 0.048434 |
|  |  |  |  | rs9892194 | A | G | 0.010602 | 0.002062 | 2.71E-07 | 0.004158 | 0.007404 | 0.574435 |
|  |  |  |  | rs9946771 | T | C | -0.0234 | 0.004077 | 9.47E-09 | 0.009057 | 0.013459 | 0.500991 |
| Vitamin E | | Bacterial infection | 23.04 | rs111306778 | A | G | -0.04797 | 0.009572 | 5.42E-07 | -0.01184 | 0.011941 | 0.321322 |
|  |  |  |  | rs12421920 | G | A | -0.04345 | 0.009385 | 3.67E-06 | 0.013334 | 0.016601 | 0.421873 |
|  |  |  |  | rs12899673 | A | G | 0.026895 | 0.005818 | 3.79E-06 | 0.004545 | 0.007563 | 0.547854 |
|  |  |  |  | rs35218694 | G | A | -0.07416 | 0.01531 | 1.27E-06 | 0.002577 | 0.025625 | 0.919908 |
|  |  |  |  | rs6033 | G | A | -0.05166 | 0.010556 | 9.91E-07 | -0.01626 | 0.011488 | 0.156885 |
|  |  |  |  | rs71385328 | G | A | 0.130015 | 0.026206 | 7.01E-07 | 0.011391 | 0.015595 | 0.465161 |
|  |  |  |  | rs79966958 | T | C | -0.11653 | 0.024505 | 1.98E-06 | 0.012142 | 0.027289 | 0.656379 |
|  |  |  |  | rs979218 | C | A | -0.04303 | 0.009223 | 3.07E-06 | 0.00593 | 0.009895 | 0.548973 |
| Bacterial infection | | Vitamin A | 27.969 | rs10819189 | A | G | -0.0438 | 0.009568 | 4.69E-06 | 0.025166 | 0.017074 | 0.140509 |
|  |  |  |  | rs10874332 | C | T | 0.03491 | 0.007417 | 2.52E-06 | -0.03086 | 0.015735 | 0.049891 |
|  |  |  |  | rs12471458 | G | A | -0.04756 | 0.010256 | 3.53E-06 | 0.024646 | 0.020567 | 0.230777 |
|  |  |  |  | rs142277318 | T | G | 0.130114 | 0.025024 | 2.00E-07 | -0.02717 | 0.075037 | 0.717237 |
|  |  |  |  | rs1523691 | A | G | -0.03714 | 0.007311 | 3.77E-07 | 0.004436 | 0.01525 | 0.771143 |
|  |  |  |  | rs1800947 | G | C | -0.12872 | 0.015642 | 1.89E-16 | -0.06401 | 0.032966 | 0.052186 |
|  |  |  |  | rs1846959 | C | T | -0.03879 | 0.008103 | 1.69E-06 | 0.009136 | 0.017805 | 0.607889 |
|  |  |  |  | rs35560006 | G | A | -0.04933 | 0.009872 | 5.81E-07 | 0.003656 | 0.020738 | 0.86008 |
|  |  |  |  | rs429358 | C | T | -0.06437 | 0.009596 | 1.97E-11 | 0.022982 | 0.021883 | 0.293619 |
|  |  |  |  | rs602457 | C | T | 0.081028 | 0.010116 | 1.15E-15 | 0.01301 | 0.020092 | 0.517298 |
|  |  |  |  | rs62488178 | C | G | 0.039616 | 0.008418 | 2.53E-06 | -0.00328 | 0.018474 | 0.858907 |
|  |  |  |  | rs7085082 | C | T | 0.035721 | 0.007407 | 1.42E-06 | 0.003164 | 0.015524 | 0.838506 |
|  |  |  |  | rs71659344 | T | A | -0.11222 | 0.022082 | 3.73E-07 | -0.06254 | 0.072247 | 0.386654 |
|  |  |  |  | rs7282182 | T | C | -0.03442 | 0.007324 | 2.61E-06 | 0.010197 | 0.01551 | 0.510877 |
|  |  |  |  | rs73011245 | A | G | 0.046608 | 0.010039 | 3.44E-06 | 0.047471 | 0.024998 | 0.057567 |
|  |  |  |  | rs73224019 | A | C | -0.04948 | 0.010373 | 1.84E-06 | 0.02621 | 0.021342 | 0.2194 |
|  |  |  |  | rs74987515 | G | C | 0.243787 | 0.05261 | 3.59E-06 | 0.085361 | 0.09402 | 0.363929 |
|  |  |  |  | rs76592164 | A | G | 0.097552 | 0.021357 | 4.93E-06 | 0.051963 | 0.036792 | 0.157848 |
|  |  |  |  | rs77782776 | T | C | 0.066887 | 0.013314 | 5.07E-07 | -0.01326 | 0.033096 | 0.68874 |
|  |  |  |  | rs78997994 | A | G | -0.26726 | 0.057652 | 3.56E-06 | -0.03673 | 0.059528 | 0.537239 |
|  |  |  |  | rs9983131 | G | T | 0.064852 | 0.013703 | 2.21E-06 | -0.00825 | 0.030114 | 0.784146 |
| Bacterial infection | | Vitamin B6 |  | rs10789396 | C | T | -0.03574 | 0.007297 | 9.66E-07 | 0.008688 | 0.005777 | 0.13 |
|  |  |  |  | rs10819189 | A | G | -0.0438 | 0.009568 | 4.69E-06 | 0.012908 | 0.006054 | 0.033 |
|  |  |  |  | rs10874332 | C | T | 0.03491 | 0.007417 | 2.52E-06 | -0.00339 | 0.00557 | 0.54 |
|  |  |  |  | rs12471458 | G | A | -0.04756 | 0.010256 | 3.53E-06 | -0.00427 | 0.007457 | 0.57 |
|  |  |  |  | rs142277318 | T | G | 0.130114 | 0.025024 | 2.00E-07 | -0.04171 | 0.025537 | 0.1 |
|  |  |  |  | rs1523691 | A | G | -0.03714 | 0.007311 | 3.77E-07 | 0.006412 | 0.005429 | 0.24 |
|  |  |  |  | rs1800947 | G | C | -0.12872 | 0.015642 | 1.89E-16 | -0.00651 | 0.011503 | 0.57 |
|  |  |  |  | rs1846959 | C | T | -0.03879 | 0.008103 | 1.69E-06 | -0.003 | 0.006153 | 0.630001 |
|  |  |  |  | rs35560006 | G | A | -0.04933 | 0.009872 | 5.81E-07 | -0.00287 | 0.00748 | 0.7 |
|  |  |  |  | rs429358 | C | T | -0.06437 | 0.009596 | 1.97E-11 | 0.001845 | 0.007617 | 0.81 |
|  |  |  |  | rs602457 | C | T | 0.081028 | 0.010116 | 1.15E-15 | 0.006584 | 0.007496 | 0.38 |
|  |  |  |  | rs62488178 | C | G | 0.039616 | 0.008418 | 2.53E-06 | -0.00847 | 0.006622 | 0.2 |
|  |  |  |  | rs7085082 | C | T | 0.035721 | 0.007407 | 1.42E-06 | 0.002484 | 0.005446 | 0.649999 |
|  |  |  |  | rs71659344 | T | A | -0.11222 | 0.022082 | 3.73E-07 | 0.001803 | 0.02482 | 0.94 |
|  |  |  |  | rs7282182 | T | C | -0.03442 | 0.007324 | 2.61E-06 | 0.007229 | 0.00551 | 0.19 |
|  |  |  |  | rs73011245 | A | G | 0.046608 | 0.010039 | 3.44E-06 | 0.001799 | 0.008628 | 0.83 |
|  |  |  |  | rs73224019 | A | C | -0.04948 | 0.010373 | 1.84E-06 | -0.00296 | 0.007786 | 0.7 |
|  |  |  |  | rs74987515 | G | C | 0.243787 | 0.05261 | 3.59E-06 | 0.049665 | 0.035473 | 0.16 |
|  |  |  |  | rs76592164 | A | G | 0.097552 | 0.021357 | 4.93E-06 | -0.01734 | 0.013068 | 0.18 |
|  |  |  |  | rs77782776 | T | C | 0.066887 | 0.013314 | 5.07E-07 | 0.004636 | 0.011916 | 0.7 |
|  |  |  |  | rs78997994 | A | G | -0.26726 | 0.057652 | 3.56E-06 | -0.0288 | 0.020736 | 0.16 |
|  |  |  |  | rs9983131 | G | T | 0.064852 | 0.013703 | 2.21E-06 | -0.00537 | 0.011063 | 0.630001 |
| Bacterial infection | | Vitamin B12 |  | rs10789396 | C | T | -0.03574 | 0.007297 | 9.66E-07 | -0.00073 | 0.0058 | 0.9 |
|  |  |  |  | rs10819189 | A | G | -0.0438 | 0.009568 | 4.69E-06 | 0.001484 | 0.006077 | 0.81 |
|  |  |  |  | rs10874332 | C | T | 0.03491 | 0.007417 | 2.52E-06 | -0.01112 | 0.005592 | 0.047 |
|  |  |  |  | rs12471458 | G | A | -0.04756 | 0.010256 | 3.53E-06 | -0.00909 | 0.007487 | 0.22 |
|  |  |  |  | rs142277318 | T | G | 0.130114 | 0.025024 | 2.00E-07 | -0.01734 | 0.025637 | 0.5 |
|  |  |  |  | rs1523691 | A | G | -0.03714 | 0.007311 | 3.77E-07 | 0.010956 | 0.005449 | 0.044 |
|  |  |  |  | rs1800947 | G | C | -0.12872 | 0.015642 | 1.89E-16 | 0.006934 | 0.011548 | 0.55 |
|  |  |  |  | rs1846959 | C | T | -0.03879 | 0.008103 | 1.69E-06 | -0.01005 | 0.006176 | 0.1 |
|  |  |  |  | rs35560006 | G | A | -0.04933 | 0.009872 | 5.81E-07 | -0.00187 | 0.007509 | 0.8 |
|  |  |  |  | rs429358 | C | T | -0.06437 | 0.009596 | 1.97E-11 | -0.00296 | 0.007647 | 0.7 |
|  |  |  |  | rs602457 | C | T | 0.081028 | 0.010116 | 1.15E-15 | -0.00761 | 0.007525 | 0.31 |
|  |  |  |  | rs62488178 | C | G | 0.039616 | 0.008418 | 2.53E-06 | -0.00192 | 0.006647 | 0.77 |
|  |  |  |  | rs7085082 | C | T | 0.035721 | 0.007407 | 1.42E-06 | -0.0059 | 0.005467 | 0.28 |
|  |  |  |  | rs71659344 | T | A | -0.11222 | 0.022082 | 3.73E-07 | -0.02106 | 0.024917 | 0.4 |
|  |  |  |  | rs7282182 | T | C | -0.03442 | 0.007324 | 2.61E-06 | 0.001273 | 0.005531 | 0.82 |
|  |  |  |  | rs73011245 | A | G | 0.046608 | 0.010039 | 3.44E-06 | -0.00504 | 0.008662 | 0.56 |
|  |  |  |  | rs73224019 | A | C | -0.04948 | 0.010373 | 1.84E-06 | -0.00019 | 0.007815 | 0.98 |
|  |  |  |  | rs74987515 | G | C | 0.243787 | 0.05261 | 3.59E-06 | 0.026784 | 0.035612 | 0.450001 |
|  |  |  |  | rs76592164 | A | G | 0.097552 | 0.021357 | 4.93E-06 | -0.01117 | 0.013118 | 0.39 |
|  |  |  |  | rs77782776 | T | C | 0.066887 | 0.013314 | 5.07E-07 | 0.026996 | 0.011964 | 0.024 |
|  |  |  |  | rs78997994 | A | G | -0.26726 | 0.057652 | 3.56E-06 | 0.016103 | 0.020816 | 0.44 |
|  |  |  |  | rs9983131 | G | T | 0.064852 | 0.013703 | 2.21E-06 | -0.01541 | 0.011105 | 0.17 |
| Bacterial infection | | Vitamin C |  | rs10789396 | C | T | -0.03574 | 0.007297 | 9.66E-07 | 0.004653 | 0.00583 | 0.42 |
|  |  |  |  | rs10874332 | C | T | 0.03491 | 0.007417 | 2.52E-06 | 0.008915 | 0.005621 | 0.11 |
|  |  |  |  | rs12471458 | G | A | -0.04756 | 0.010256 | 3.53E-06 | -0.001 | 0.007524 | 0.89 |
|  |  |  |  | rs142277318 | T | G | 0.130114 | 0.025024 | 2.00E-07 | -0.0506 | 0.02577 | 0.05 |
|  |  |  |  | rs1800947 | G | C | -0.12872 | 0.015642 | 1.89E-16 | 0.005498 | 0.011608 | 0.64 |
|  |  |  |  | rs1846959 | C | T | -0.03879 | 0.008103 | 1.69E-06 | -0.00711 | 0.006209 | 0.25 |
|  |  |  |  | rs35560006 | G | A | -0.04933 | 0.009872 | 5.81E-07 | -0.00598 | 0.007549 | 0.43 |
|  |  |  |  | rs429358 | C | T | -0.06437 | 0.009596 | 1.97E-11 | 0.011663 | 0.007688 | 0.13 |
|  |  |  |  | rs602457 | C | T | 0.081028 | 0.010116 | 1.15E-15 | 0.001839 | 0.007565 | 0.81 |
|  |  |  |  | rs62488178 | C | G | 0.039616 | 0.008418 | 2.53E-06 | 0.001717 | 0.006683 | 0.8 |
|  |  |  |  | rs7085082 | C | T | 0.035721 | 0.007407 | 1.42E-06 | 0.001398 | 0.005496 | 0.8 |
|  |  |  |  | rs71659344 | T | A | -0.11222 | 0.022082 | 3.73E-07 | 0.028886 | 0.025046 | 0.25 |
|  |  |  |  | rs7282182 | T | C | -0.03442 | 0.007324 | 2.61E-06 | 0.008278 | 0.005562 | 0.14 |
|  |  |  |  | rs73011245 | A | G | 0.046608 | 0.010039 | 3.44E-06 | 0.011375 | 0.008708 | 0.19 |
|  |  |  |  | rs73224019 | A | C | -0.04948 | 0.010373 | 1.84E-06 | 0.006369 | 0.007858 | 0.42 |
|  |  |  |  | rs74987515 | G | C | 0.243787 | 0.05261 | 3.59E-06 | 0.011094 | 0.035797 | 0.760001 |
|  |  |  |  | rs76592164 | A | G | 0.097552 | 0.021357 | 4.93E-06 | -0.00289 | 0.013189 | 0.83 |
|  |  |  |  | rs77782776 | T | C | 0.066887 | 0.013314 | 5.07E-07 | 0.009863 | 0.012024 | 0.41 |
|  |  |  |  | rs78997994 | A | G | -0.26726 | 0.057652 | 3.56E-06 | -0.03757 | 0.020927 | 0.073 |
|  |  |  |  | rs9983131 | G | T | 0.064852 | 0.013703 | 2.21E-06 | 0.015658 | 0.011166 | 0.16 |
| Bacterial infection | | Vitamin D |  | rs10789396 | C | T | -0.03574 | 0.007297 | 9.66E-07 | -0.0019 | 0.005823 | 0.74 |
|  |  |  |  | rs10819189 | A | G | -0.0438 | 0.009568 | 4.69E-06 | -0.00172 | 0.006102 | 0.780001 |
|  |  |  |  | rs10874332 | C | T | 0.03491 | 0.007417 | 2.52E-06 | -0.00759 | 0.005615 | 0.18 |
|  |  |  |  | rs12471458 | G | A | -0.04756 | 0.010256 | 3.53E-06 | 0.002609 | 0.007517 | 0.73 |
|  |  |  |  | rs142277318 | T | G | 0.130114 | 0.025024 | 2.00E-07 | -0.04914 | 0.025742 | 0.056 |
|  |  |  |  | rs1523691 | A | G | -0.03714 | 0.007311 | 3.77E-07 | 0.008991 | 0.005472 | 0.1 |
|  |  |  |  | rs1800947 | G | C | -0.12872 | 0.015642 | 1.89E-16 | 0.001078 | 0.011595 | 0.93 |
|  |  |  |  | rs1846959 | C | T | -0.03879 | 0.008103 | 1.69E-06 | -0.00485 | 0.006201 | 0.43 |
|  |  |  |  | rs35560006 | G | A | -0.04933 | 0.009872 | 5.81E-07 | -0.00933 | 0.007539 | 0.22 |
|  |  |  |  | rs429358 | C | T | -0.06437 | 0.009596 | 1.97E-11 | -0.013 | 0.007677 | 0.091 |
|  |  |  |  | rs602457 | C | T | 0.081028 | 0.010116 | 1.15E-15 | -0.00161 | 0.007555 | 0.83 |
|  |  |  |  | rs62488178 | C | G | 0.039616 | 0.008418 | 2.53E-06 | -0.00885 | 0.006675 | 0.18 |
|  |  |  |  | rs7085082 | C | T | 0.035721 | 0.007407 | 1.42E-06 | -0.01082 | 0.005488 | 0.049 |
|  |  |  |  | rs71659344 | T | A | -0.11222 | 0.022082 | 3.73E-07 | -0.03532 | 0.025018 | 0.16 |
|  |  |  |  | rs7282182 | T | C | -0.03442 | 0.007324 | 2.61E-06 | -0.00613 | 0.005554 | 0.27 |
|  |  |  |  | rs73011245 | A | G | 0.046608 | 0.010039 | 3.44E-06 | -0.01025 | 0.008697 | 0.24 |
|  |  |  |  | rs73224019 | A | C | -0.04948 | 0.010373 | 1.84E-06 | -0.0039 | 0.007847 | 0.62 |
|  |  |  |  | rs74987515 | G | C | 0.243787 | 0.05261 | 3.59E-06 | 0.017866 | 0.035757 | 0.62 |
|  |  |  |  | rs76592164 | A | G | 0.097552 | 0.021357 | 4.93E-06 | -0.00451 | 0.013171 | 0.73 |
|  |  |  |  | rs77782776 | T | C | 0.066887 | 0.013314 | 5.07E-07 | 0.011417 | 0.012012 | 0.34 |
|  |  |  |  | rs78997994 | A | G | -0.26726 | 0.057652 | 3.56E-06 | 0.00769 | 0.020899 | 0.709999 |
|  |  |  |  | rs9983131 | G | T | 0.064852 | 0.013703 | 2.21E-06 | -0.00314 | 0.01115 | 0.780001 |
| Bacterial infection | | 25(OH)D |  | rs10819189 | A | G | -0.0438 | 0.009568 | 4.69E-06 | -0.00085 | 0.002269 | 0.708891 |
|  |  |  |  | rs10874332 | C | T | 0.03491 | 0.007417 | 2.52E-06 | 0.002027 | 0.002076 | 0.328818 |
|  |  |  |  | rs12471458 | G | A | -0.04756 | 0.010256 | 3.53E-06 | 0.002764 | 0.002798 | 0.323271 |
|  |  |  |  | rs142277318 | T | G | 0.130114 | 0.025024 | 2.00E-07 | -0.01227 | 0.009696 | 0.205542 |
|  |  |  |  | rs1523691 | A | G | -0.03714 | 0.007311 | 3.77E-07 | 0.000279 | 0.002039 | 0.890979 |
|  |  |  |  | rs1800947 | G | C | -0.12872 | 0.015642 | 1.89E-16 | 0.001425 | 0.004367 | 0.744193 |
|  |  |  |  | rs1846959 | C | T | -0.03879 | 0.008103 | 1.69E-06 | 0.004373 | 0.002295 | 0.056647 |
|  |  |  |  | rs35560006 | G | A | -0.04933 | 0.009872 | 5.81E-07 | -0.00119 | 0.002812 | 0.672033 |
|  |  |  |  | rs62488178 | C | G | 0.039616 | 0.008418 | 2.53E-06 | -0.00665 | 0.002488 | 0.007535 |
|  |  |  |  | rs7085082 | C | T | 0.035721 | 0.007407 | 1.42E-06 | -0.00024 | 0.002034 | 0.904795 |
|  |  |  |  | rs71659344 | T | A | -0.11222 | 0.022082 | 3.73E-07 | 0.002787 | 0.009546 | 0.770327 |
|  |  |  |  | rs7282182 | T | C | -0.03442 | 0.007324 | 2.61E-06 | 0.002149 | 0.002072 | 0.299753 |
|  |  |  |  | rs73011245 | A | G | 0.046608 | 0.010039 | 3.44E-06 | -0.0021 | 0.003245 | 0.516945 |
|  |  |  |  | rs73224019 | A | C | -0.04948 | 0.010373 | 1.84E-06 | 0.00299 | 0.002905 | 0.303212 |
|  |  |  |  | rs76592164 | A | G | 0.097552 | 0.021357 | 4.93E-06 | -0.01192 | 0.004978 | 0.01664 |
|  |  |  |  | rs9983131 | G | T | 0.064852 | 0.013703 | 2.21E-06 | -0.00392 | 0.004155 | 0.345595 |
| Bacterial infection | | Vitamin E |  | rs10789396 | C | T | -0.03574 | 0.007297 | 9.66E-07 | 0.005022 | 0.005821 | 0.39 |
|  |  |  |  | rs10819189 | A | G | -0.0438 | 0.009568 | 4.69E-06 | 0.017087 | 0.006101 | 0.0051 |
|  |  |  |  | rs10874332 | C | T | 0.03491 | 0.007417 | 2.52E-06 | 0.00312 | 0.005613 | 0.58 |
|  |  |  |  | rs12471458 | G | A | -0.04756 | 0.010256 | 3.53E-06 | 0.009944 | 0.007515 | 0.19 |
|  |  |  |  | rs142277318 | T | G | 0.130114 | 0.025024 | 2.00E-07 | -0.03674 | 0.025734 | 0.15 |
|  |  |  |  | rs1523691 | A | G | -0.03714 | 0.007311 | 3.77E-07 | 0.006904 | 0.005471 | 0.21 |
|  |  |  |  | rs1800947 | G | C | -0.12872 | 0.015642 | 1.89E-16 | -0.00364 | 0.011592 | 0.75 |
|  |  |  |  | rs1846959 | C | T | -0.03879 | 0.008103 | 1.69E-06 | -0.00654 | 0.006201 | 0.29 |
|  |  |  |  | rs35560006 | G | A | -0.04933 | 0.009872 | 5.81E-07 | -0.01817 | 0.007539 | 0.016 |
|  |  |  |  | rs429358 | C | T | -0.06437 | 0.009596 | 1.97E-11 | -0.00392 | 0.007677 | 0.61 |
|  |  |  |  | rs602457 | C | T | 0.081028 | 0.010116 | 1.15E-15 | 5.49E-05 | 0.007555 | 0.99 |
|  |  |  |  | rs62488178 | C | G | 0.039616 | 0.008418 | 2.53E-06 | 0.001042 | 0.006674 | 0.88 |
|  |  |  |  | rs7085082 | C | T | 0.035721 | 0.007407 | 1.42E-06 | 0.001568 | 0.005488 | 0.780001 |
|  |  |  |  | rs71659344 | T | A | -0.11222 | 0.022082 | 3.73E-07 | 0.004464 | 0.025011 | 0.86 |
|  |  |  |  | rs7282182 | T | C | -0.03442 | 0.007324 | 2.61E-06 | 0.009358 | 0.005553 | 0.092 |
|  |  |  |  | rs73011245 | A | G | 0.046608 | 0.010039 | 3.44E-06 | -0.01382 | 0.008696 | 0.11 |
|  |  |  |  | rs73224019 | A | C | -0.04948 | 0.010373 | 1.84E-06 | 0.000176 | 0.007847 | 0.98 |
|  |  |  |  | rs74987515 | G | C | 0.243787 | 0.05261 | 3.59E-06 | -0.04716 | 0.035747 | 0.19 |
|  |  |  |  | rs76592164 | A | G | 0.097552 | 0.021357 | 4.93E-06 | 0.002438 | 0.01317 | 0.85 |
|  |  |  |  | rs77782776 | T | C | 0.066887 | 0.013314 | 5.07E-07 | 0.004062 | 0.01201 | 0.74 |
|  |  |  |  | rs78997994 | A | G | -0.26726 | 0.057652 | 3.56E-06 | -0.03522 | 0.020898 | 0.092 |
|  |  |  |  | rs9983131 | G | T | 0.064852 | 0.013703 | 2.21E-06 | -0.00176 | 0.01115 | 0.87 |
