# Supplementary material for: Causality of circulating vitamins on infectious diseases: integrating Mendelian randomization and in vivo evidence
Source: Front Immunol. 2025 Dec 1;16:1674678. doi: 10.3389/fimmu.2025.1674678 (PMC12702853; doi:10.3389/fimmu.2025.1674678)
Supplement: Supplementary file 2 [file Table2.docx]

| Exposure | Outcome | Pleiotropy test (MR-Egger) | | |
| --- | --- | --- | --- | --- |
|  |  | Intercept | se | p |
| Vitamin A | Viral infection | 0.01419268 | 0.01301516 | 0.3010832 |
| Vitamin B6 | Viral infection | 0.009667275 | 0.01472505 | 0.522136 |
| Vitamin B12 | Viral infection | 0.01110078 | 0.02032569 | 0.6046633 |
| Vitamin C | Viral infection | -0.004986573 | 0.01276664 | 0.7062925 |
| Vitamin D | Viral infection | -0.02004974 | 0.02396825 | 0.422406 |
| 25(OH)D | Viral infection | 0.002818638 | 0.001136026 | 0.01395598 |
| Vitamin E | Viral infection | 0.001931402 | 0.01226876 | 0.8780436 |
| Viral infection | Vitamin A | 0.01782822 | 0.02590153 | 0.5169733 |
| Viral infection | Vitamin B6 | -0.005511643 | 0.01006974 | 0.6038872 |
| Viral infection | Vitamin B12 | -0.007553727 | 0.007731324 | 0.3662874 |
| Viral infection | Vitamin C | -0.01584381 | 0.007771562 | 0.0876031 |
| Viral infection | Vitamin D | 0.002069411 | 0.007762608 | 0.7987123 |
| Viral infection | 25(OH)D | 0.000715516 | 0.006019149 | 0.9128889 |
| Viral infection | Vitamin E | -0.005738022 | 0.00991679 | 0.5838983 |
| Vitamin A | Bacterial infection | 0.007612786 | 0.008703722 | 0.4022779 |
| Vitamin B6 | Bacterial infection | 0.002914991 | 0.006872501 | 0.6778995 |
| Vitamin B12 | Bacterial infection | 0.004784881 | 0.01016111 | 0.6543436 |
| Vitamin C | Bacterial infection | -0.000380736 | 0.004065635 | 0.9264238 |
| Vitamin D | Bacterial infection | -0.002411731 | 0.003762667 | 0.5288235 |
| 25(OH)D | Bacterial infection | 0.002299149 | 0.002052493 | 0.264039 |
| Vitamin E | Bacterial infection | 0.003151138 | 0.009595035 | 0.7493726 |
| Bacterial infection | Vitamin A | -0.02704242 | 0.009814896 | 0.012591 |
| Bacterial infection | Vitamin B6 | -0.008062848 | 0.003350465 | 0.02589271 |
| Bacterial infection | Vitamin B12 | -0.001523721 | 0.003672741 | 0.6826514 |
| Bacterial infection | Vitamin C | -0.004148583 | 0.004458512 | 0.3632158 |
| Bacterial infection | Vitamin D | -0.002411731 | 0.003762667 | 0.5288235 |
| Bacterial infection | 25(OH)D | -0.001273241 | 0.004298131 | 0.7711154 |
| Bacterial infection | Vitamin E | -0.005168209 | 0.004079479 | 0.2197534 |
