# Supplementary material for: Causality of circulating vitamins on infectious diseases: integrating Mendelian randomization and in vivo evidence
Source: Front Immunol. 2025 Dec 1;16:1674678. doi: 10.3389/fimmu.2025.1674678 (PMC12702853; doi:10.3389/fimmu.2025.1674678)
Supplement: Supplementary file 3 [file Table3.docx]

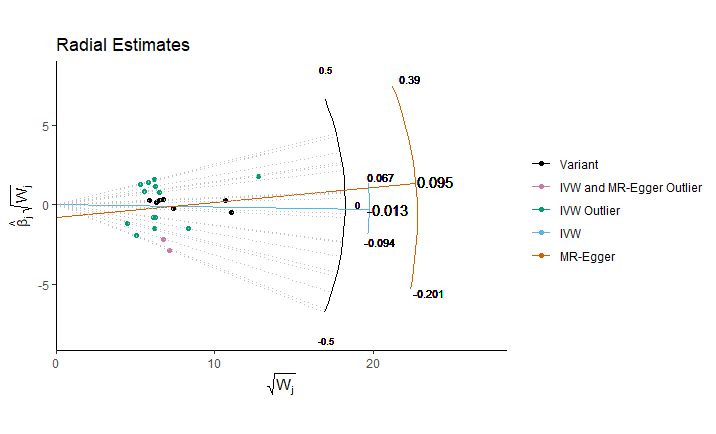


Exposure：Bacterial infection; Outcome: Vitamin C.


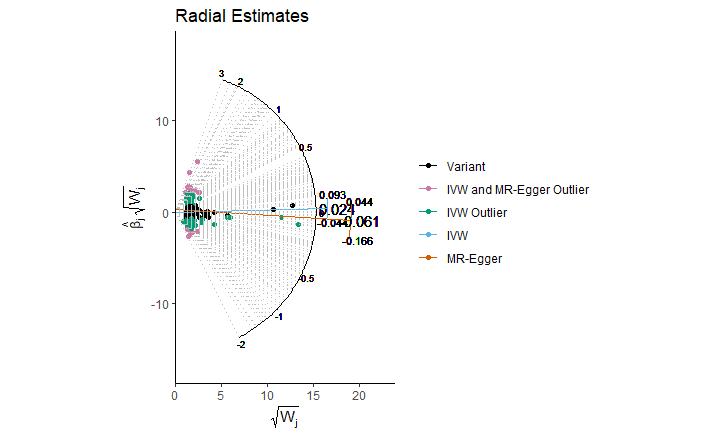


Exposure：25(OH)D; Outcome: Bacterial infection.


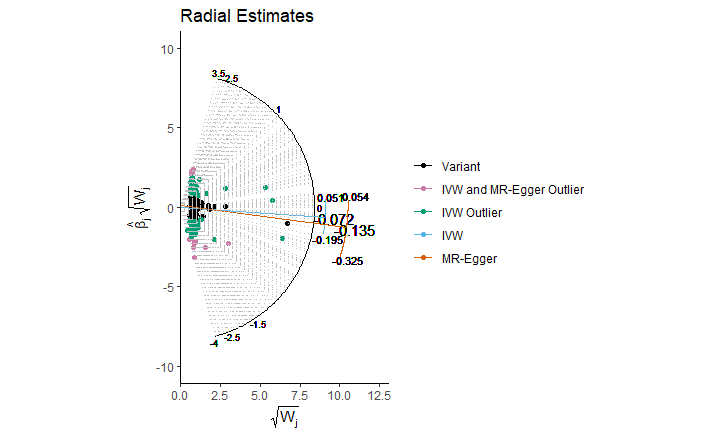


Exposure：25(OH)D; Outcome: Viral infection.


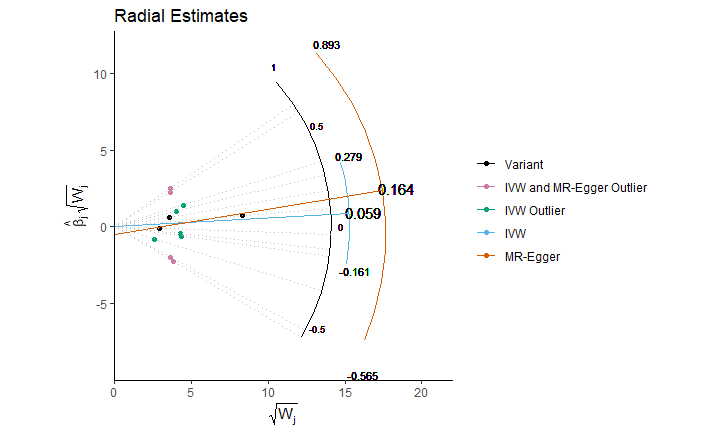


Exposure：Vitamin E; Outcome: Bacterial infection.
