# Supplementary material for: Causality of circulating vitamins on infectious diseases: integrating Mendelian randomization and in vivo evidence
Source: Front Immunol. 2025 Dec 1;16:1674678. doi: 10.3389/fimmu.2025.1674678 (PMC12702853; doi:10.3389/fimmu.2025.1674678)
Supplement: Supplementary file 4 [file Table4.docx]

| Exposure | Outcome | MR-PRESSO | | RadialMR（N of Outlier SNPs） |
| --- | --- | --- | --- | --- |
|  |  | Global Test（Pvalue） | Distortion Test（Pvalue） |  |
| Vitamin A | Viral infection | 0.7802 |  |  |
| Vitamin B6 | Viral infection | 0.2562 |  |  |
| Vitamin B12 | Viral infection | 0.7371 |  |  |
| Vitamin C | Viral infection | 0.6632 |  |  |
| Vitamin D | Viral infection | 0.523 |  |  |
| 25(OH)D | Viral infection | 0.0203 |  | 14 |
| Vitamin E | Viral infection | 0.7991 |  |  |
| Viral infection | Vitamin A | 0.2307 |  |  |
| Viral infection | Vitamin B6 | 0.19 |  |  |
| Viral infection | Vitamin B12 | 0.8932 |  |  |
| Viral infection | Vitamin C | 0.2701 |  |  |
| Viral infection | Vitamin D | 0.7694 |  |  |
| Viral infection | 25(OH)D | 0.2814 |  |  |
| Viral infection | Vitamin E | 0.2341 |  |  |
| Vitamin A | Bacterial infection | 0.0704 |  |  |
| Vitamin B6 | Bacterial infection | 0.4271 |  |  |
| Vitamin B12 | Bacterial infection | 0.9503 |  |  |
| Vitamin C | Bacterial infection | 0.0699 |  |  |
| Vitamin D | Bacterial infection | 0.6265 |  |  |
| 25(OH)D | Bacterial infection | <1e-04 | 0.264 | 19 |
| Vitamin E | Bacterial infection | 0.0173 |  | 4 |
| Bacterial infection | Vitamin A | 0.2845 |  |  |
| Bacterial infection | Vitamin B6 | 0.3398 |  |  |
| Bacterial infection | Vitamin B12 | 0.3228 |  |  |
| Bacterial infection | Vitamin C | 0.0214 |  | 2 |
| Bacterial infection | Vitamin D | 0.2643 |  |  |
| Bacterial infection | 25(OH)D | <1e-04 | 0.5193 |  |
| Bacterial infection | Vitamin E | 0.0629 |  |  |
