# Supplementary material for: Causality of circulating vitamins on infectious diseases: integrating Mendelian randomization and in vivo evidence
Source: Front Immunol. 2025 Dec 1;16:1674678. doi: 10.3389/fimmu.2025.1674678 (PMC12702853; doi:10.3389/fimmu.2025.1674678)
Supplement: Supplementary file 5 [file Table5.docx]

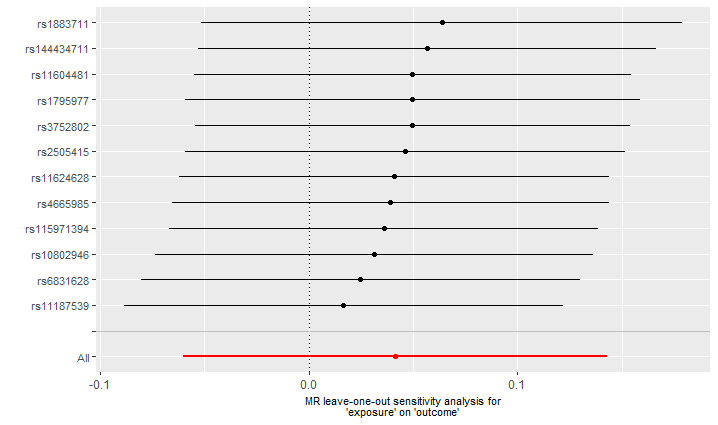


Exposure：Vitamin A; Outcome: Viral infection.


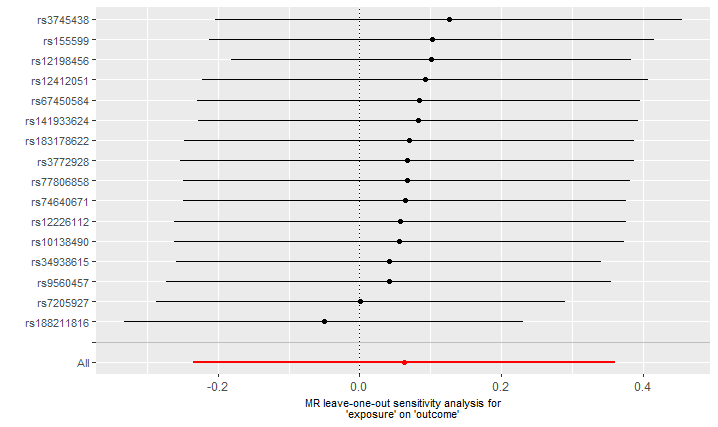


Exposure：Vitamin B6; Outcome: Viral infection.


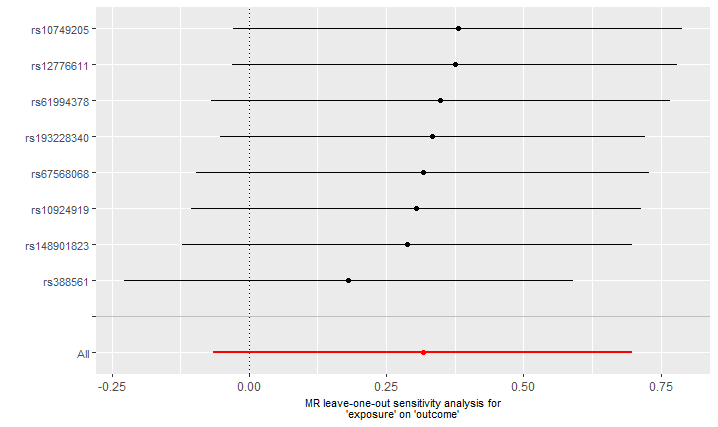


Exposure：Vitamin B12; Outcome: Viral infection.


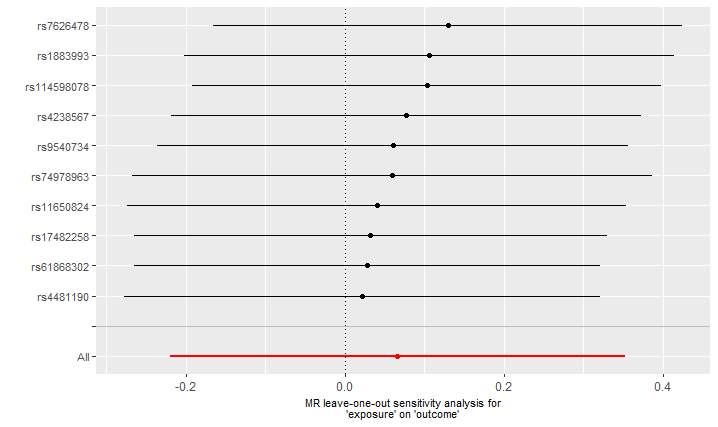


Exposure：Vitamin C; Outcome: Viral infection.


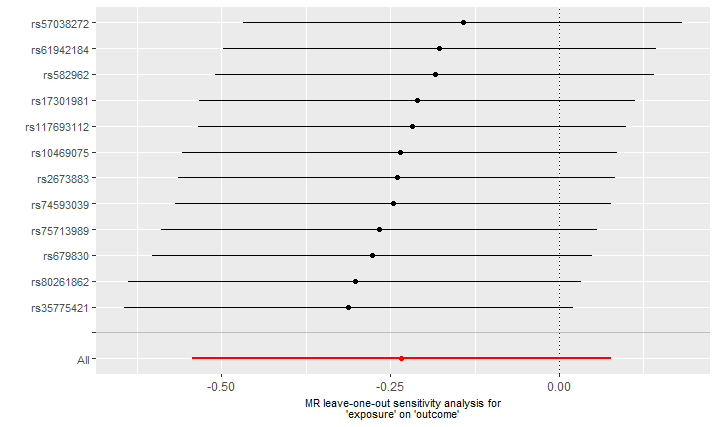


Exposure：Vitamin D; Outcome: Viral infection.


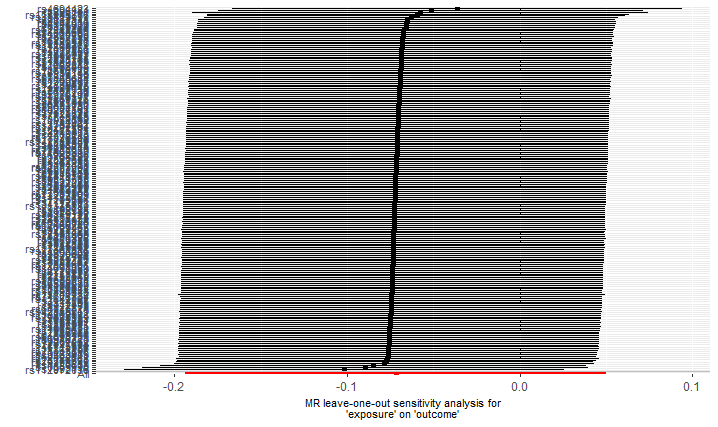


Exposure：25(OH)D; Outcome: Viral infection.


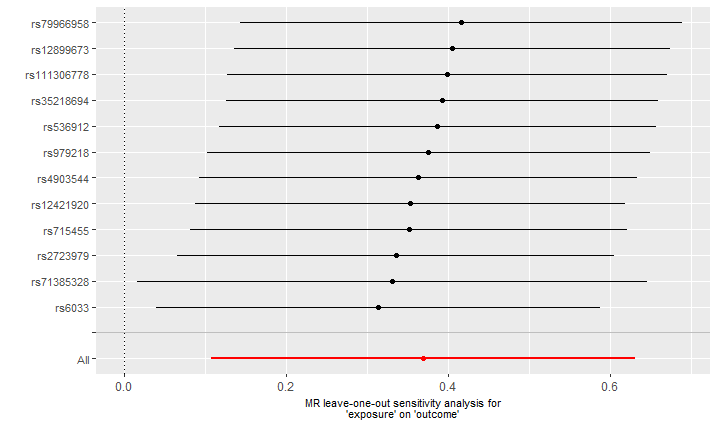


Exposure：Vitamin E; Outcome: Viral infection.


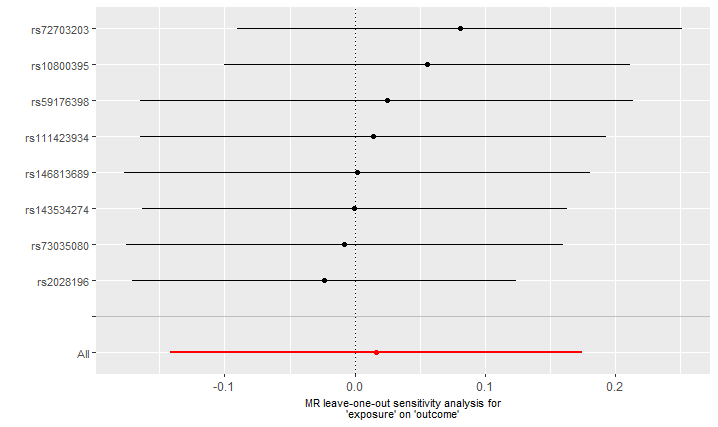


Exposure：Viral infection; Outcome: Vitamin A.


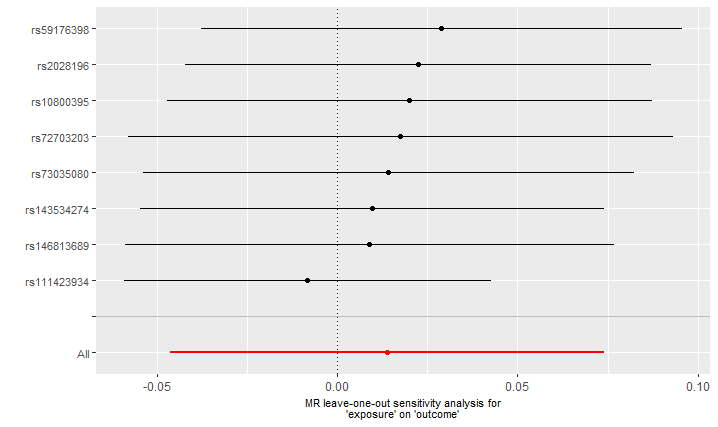


Exposure：Viral infection; Outcome: Vitamin B6.


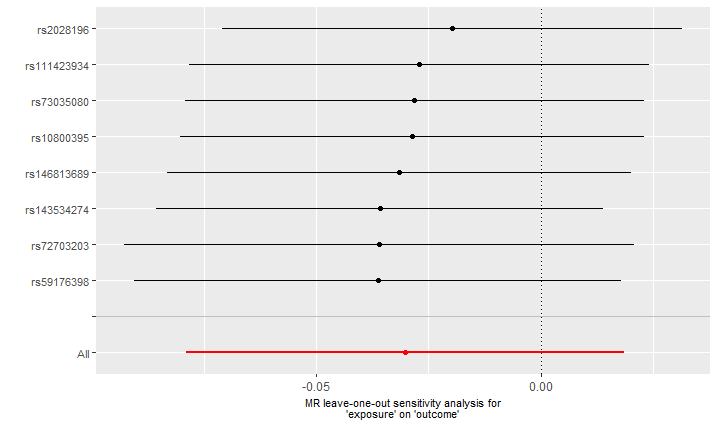


Exposure：Viral infection; Outcome: Vitamin B12.


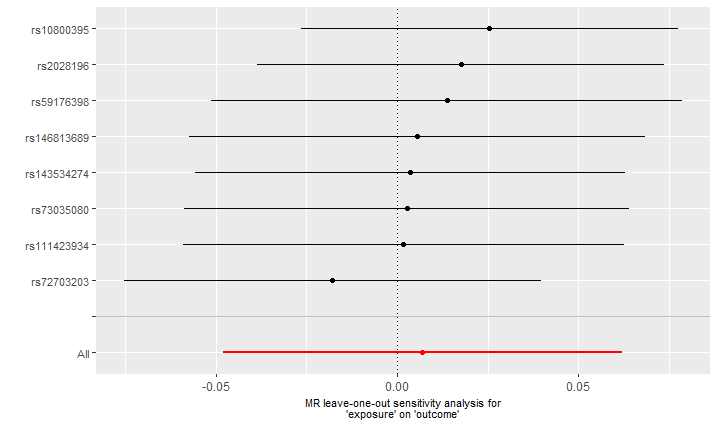


Exposure：Viral infection; Outcome: Vitamin C.


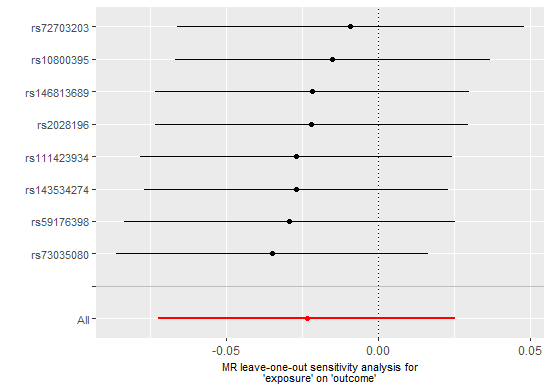


Exposure：Viral infection; Outcome: Vitamin D.


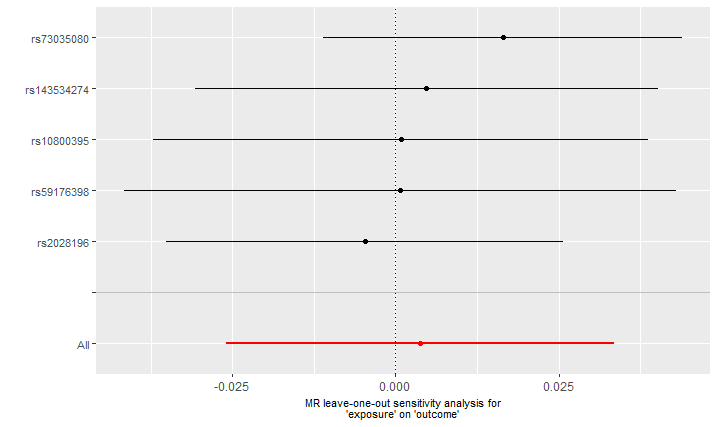


Exposure：Viral infection; Outcome:25(OH)D.


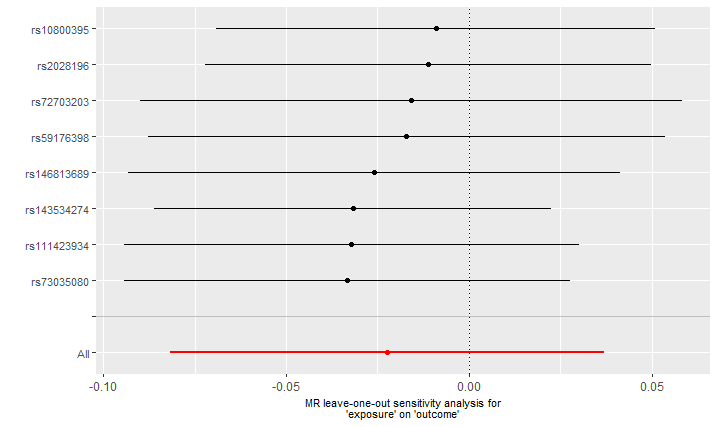


Exposure：Viral infection; Outcome: Vitamin E.


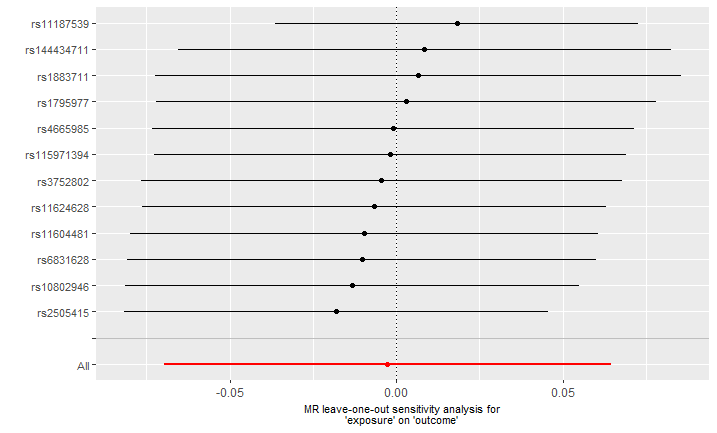


Exposure：Vitamin A; Outcome: Bacterial infection.


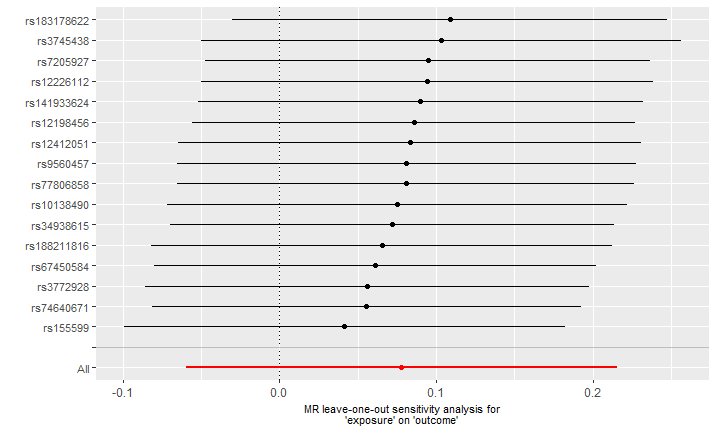


Exposure：Vitamin B6; Outcome: Bacterial infection.


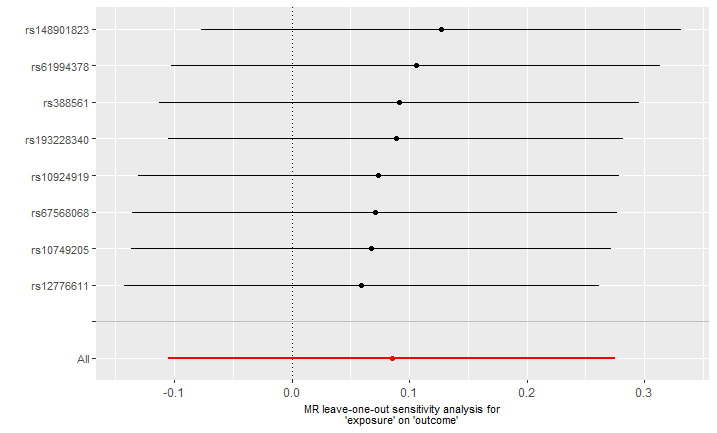


Exposure：Vitamin B12; Outcome: Bacterial infection.


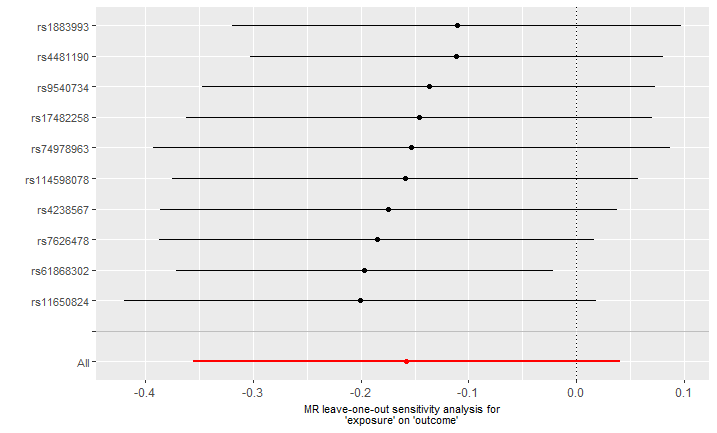


Exposure：Vitamin C; Outcome: Bacterial infection.


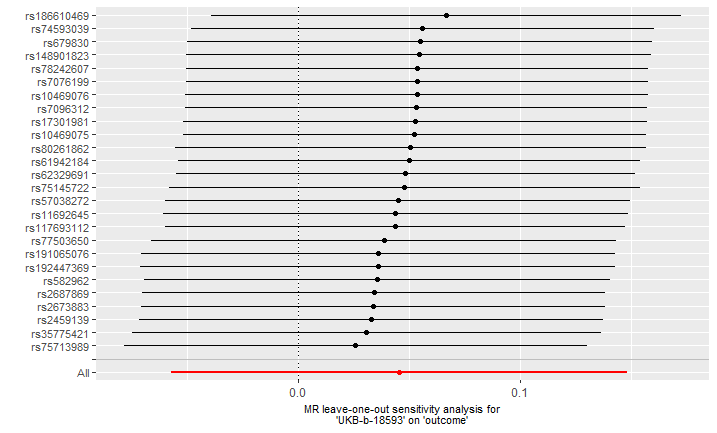


Exposure：Vitamin D; Outcome: Bacterial infection.


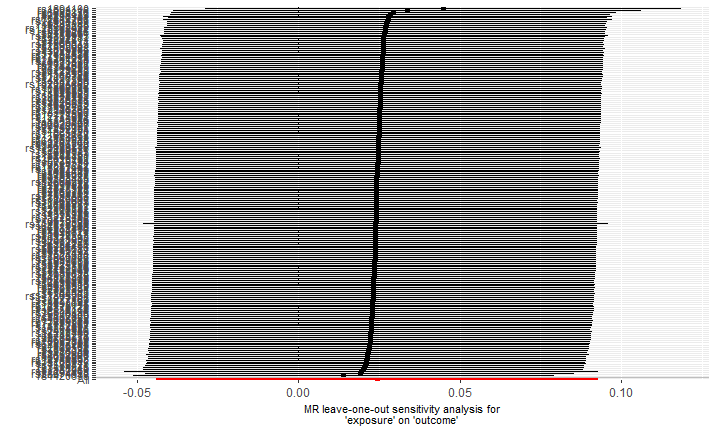


Exposure：25(OH)D; Outcome: Bacterial infection.


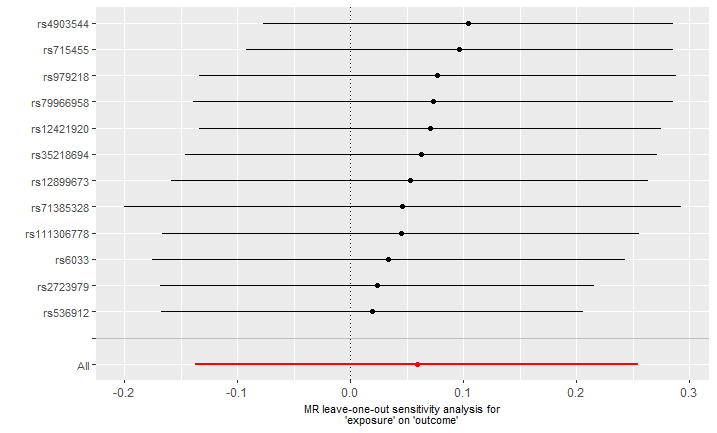


Exposure：Vitamin E; Outcome: Bacterial infection.


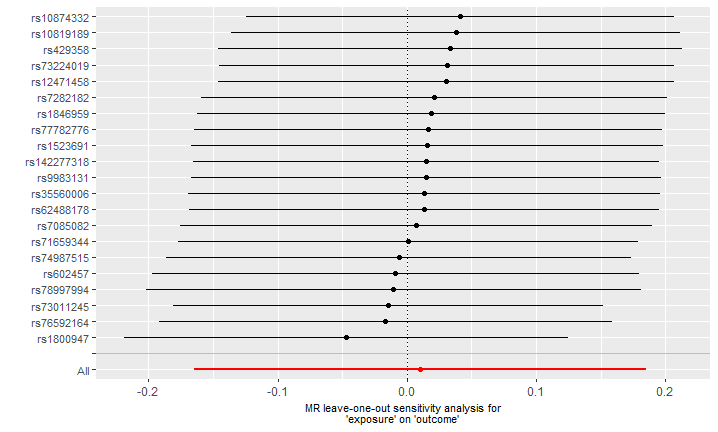


Exposure：Bacterial infection; Outcome: Vitamin A.


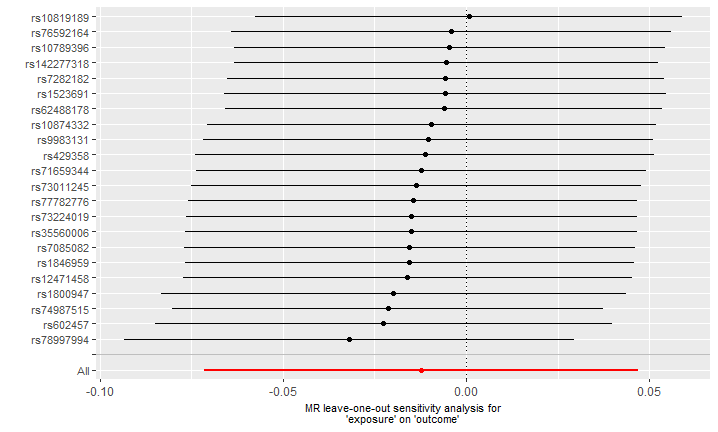


Exposure：Bacterial infection; Outcome: Vitamin B6.


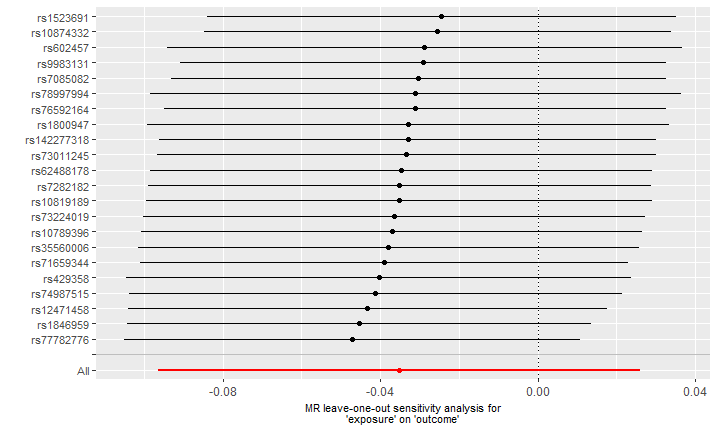


Exposure：Bacterial infection; Outcome: Vitamin B12.


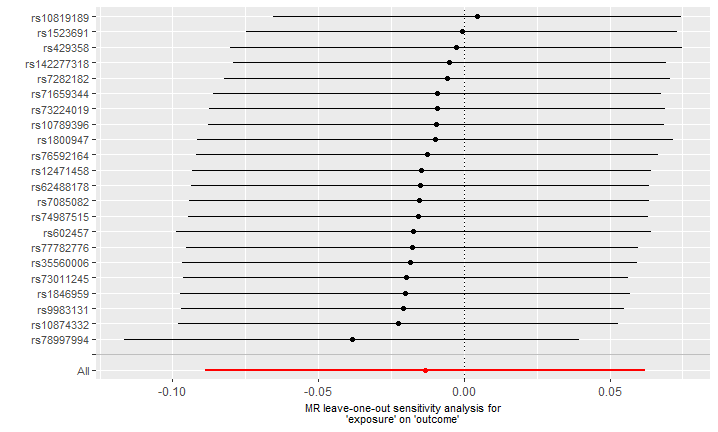


Exposure：Bacterial infection; Outcome: Vitamin C.


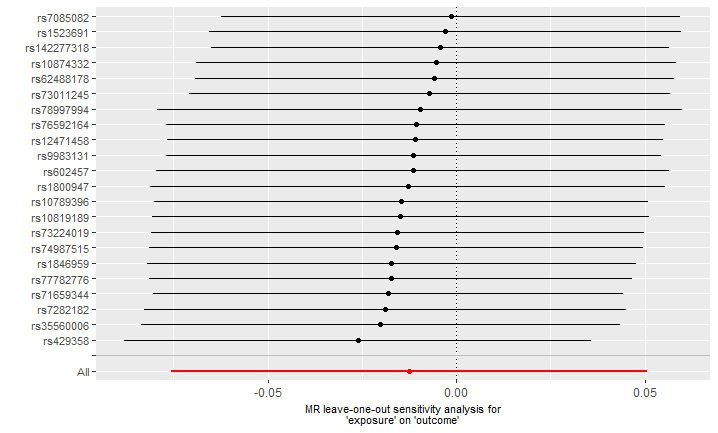


Exposure：Bacterial infection; Outcome: Vitamin D.


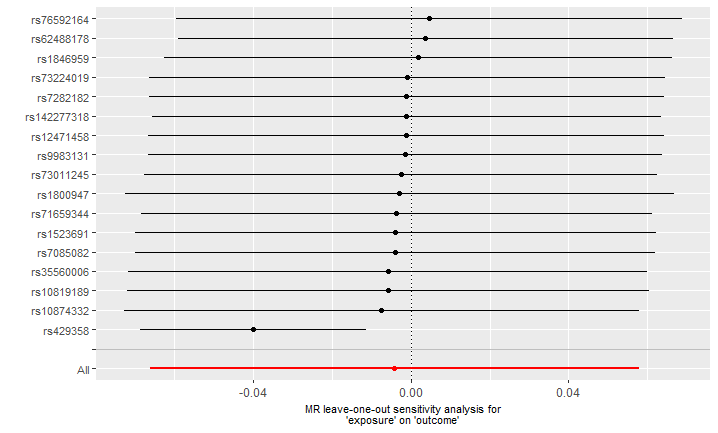


Exposure：Bacterial infection; Outcome: 25(OH)D.


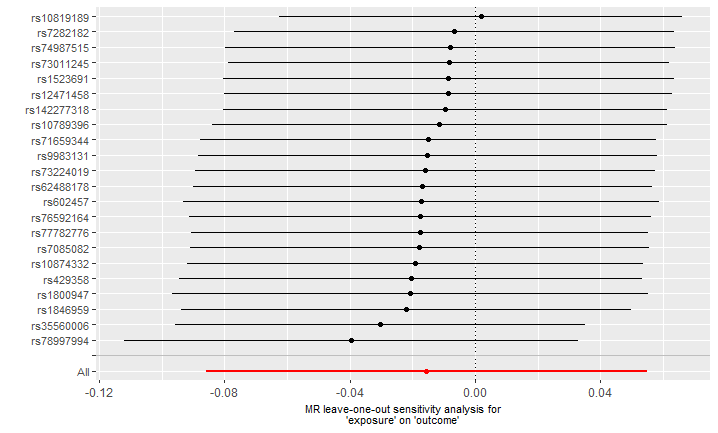


Exposure：Bacterial infection; Outcome: Vitamin E.
